# Pyrroloquinoline quinone alleviates natural aging‐related osteoporosis via a novel MCM3‐Keap1‐Nrf2 axis‐mediated stress response and Fbn1 upregulation

**DOI:** 10.1111/acel.13912

**Published:** 2023-06-26

**Authors:** Jie Li, Jing Zhang, Qi Xue, Boyang Liu, Ran Qin, Yiping Li, Yue Qiu, Rong Wang, David Goltzman, Dengshun Miao, Renlei Yang

**Affiliations:** ^1^ Department of Plastic Surgery Affiliated Friendship Plastic Surgery Hospital of Nanjing Medical University, Nanjing Medical University Nanjing China; ^2^ Department of Anatomy, Histology and Embryology, State Key Laboratory of Reproductive Medicine, The Research Center for Bone and Stem Cells Nanjing Medical University Nanjing China; ^3^ Calcium Research Laboratory McGill University Health Centre and Department of Medicine, McGill University Montreal Quebec Canada

**Keywords:** aging, Fbn1, Keap1‐Nrf2 signaling, osteoporosis, pyrroloquinoline quinone

## Abstract

Age‐related osteoporosis is associated with increased oxidative stress and cellular senescence. Pyrroloquinoline quinone (PQQ) is a water‐soluble vitamin‐like compound that has strong antioxidant capacity; however, the effect and underlying mechanism of PQQ on aging‐related osteoporosis remain unclear. The purpose of this study was to investigate whether dietary PQQ supplementation can prevent osteoporosis caused by natural aging, and the potential mechanism underlying PQQ antioxidant activity. Here, we found that when 6‐month‐old or 12‐month‐old wild‐type mice were supplemented with PQQ for 12 months or 6 months, respectively, PQQ could prevent age‐related osteoporosis in mice by inhibiting osteoclastic bone resorption and stimulating osteoblastic bone formation. Mechanistically, pharmmapper screening and molecular docking studies revealed that PQQ appears to bind to MCM3 and reduces its ubiquitination‐mediated degradation; stabilized MCM3 then competes with Nrf2 for binding to Keap1, thus activating Nrf2‐antioxidant response element (ARE) signaling. PQQ‐induced Nrf2 activation inhibited bone resorption through increasing stress response capacity and transcriptionally upregulating fibrillin‐1 (Fbn1), thus reducing Rankl production in osteoblast‐lineage cells and decreasing osteoclast activation; as well, bone formation was stimulated by inhibiting osteoblastic DNA damage and osteocyte senescence. Furthermore, Nrf2 knockout significantly blunted the inhibitory effects of PQQ on oxidative stress, on increased osteoclast activity and on the development of aging‐related osteoporosis. This study reveals the underlying mechanism of PQQ's strong antioxidant capacity and provides evidence for PQQ as a potential agent for clinical prevention and treatment of natural aging‐induced osteoporosis.

## Graphical abstract

The results of this study reveal that PQQ can bind to and stabilize MCM3, which competes with Nrf2 for binding to Keap1 and enhances Nrf2‐ARE signaling. PQQ‐induced Nrf2 activation in osteoblast‐lineage cells increases the cellular stress response and also transcriptionally activates Fbn1, thus reducing the production of Rankl and inhibiting osteoclastic bone resorption, and preventing natural aging‐related osteoporosis. In addition, PQQ‐mediated Nrf2 activation in osteoblasts‐lineage cells can inhibit DNA damage and cellular senescence and increase osteoblastic bone formation. These findings demonstrate the underlying mechanism of PQQ's strong antioxidant capacity and suggest that PQQ could be an ideal supplement for the treatment and prevention of aging‐related osteoporosis.

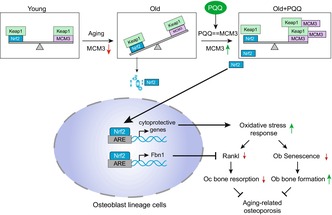

**Abbreviations**
ALT: alanine aminotransferase
ARE: antioxidant response elements
AST: aminotransferase
BMD: bone mineral density
BV/TV: bone trabecular/bone volume
ChIP: chromatin immunoprecipitation
Co‐IP: co‐immunoprecipitation
CREA: creatinine
DHE: dihydroethidium
EDTA: ethylene diamine tetraacetic acid
Fbn1: fibrillin‐1
GPx7: glutathione peroxidase7
HO1: heme oxygenase1
Keap1: Kelch‐like ECH‐associated protein 1
MAR: mineral apposition rate
MCM3: minichromosome maintenance complex component 3
MNCs: multinucleated cells
Nrf2: nuclear factor erythroid 2‐related factor 2
OB: osteoblasts
OC: osteoclasts
PGE2: prostaglandin E2
PQQ: pyrroloquinoline quinone
ROS: reactive oxygen species
SASP: senescence‐associated secretory phenotype
tBHQ: tert‐butylhydroquinone
T‐COL: total collagen
TRAP: tartrate‐resistant acid phosphatase stain
Ub‐Nrf2: ubiquitin‐Nrf2
XO: xylenol orange

## 1 INTRODUCTION

Aging has been recognized as an important risk factor for many chronic diseases, including osteoporosis. Cellular senescence, a fundamental aging mechanism implicated in multiple age‐related disorders, also drives bone loss with aging (Farr et al., 2017). Indeed, Farr and colleagues have reported that senescent cells accumulate in the bone microenvironment with aging (Farr et al., 2016), and selectively targeting senescent cells and their senescence‐associated secretory phenotype (SASP) could prevent age‐related bone loss (Farr et al., 2017). The increase in the production of ROS (Reactive Oxygen Species) with aging has long been considered a driver of cellular senescence and aging (López‐Otín et al., 2013), and maintaining redox balance has been reported to alleviate aging‐related osteoporosis (Yang, Zhang, et al., 2022).

Pyrroloquinoline‐quinine (PQQ) was first shown to function as a cofactor for oxidoreductases in bacteria (Killgore et al., 1989). PQQ‐deficient food can induce different systemic alterations in mice, including growth disorders, immune dysfunction, and abnormal reproductive capacity (Killgore et al., 1989; Steinberg et al., 1994). In contrast, PQQ supplementation has been reported to alleviate the progression of multiple diseases in mice (Dai et al., 2022; Jonscher et al., 2017). The effects of PQQ largely rely on its antioxidant function, as a previous study demonstrated that its radical‐scavenging activity is 7.4‐fold higher than that of vitamin C (Akagawa et al., 2016); however, the specific mechanism underlying its antioxidant function is still unclear. Although PQQ cannot be synthesized in mammals, it is available in foods such as milk, vegetables, and meat (Mitchell et al., 1999; Noji et al., 2007), making it an ideal regent for alleviating oxidative stress‐induced diseases, including osteoporosis. Indeed, we have previously reported that PQQ supplementation can alleviate estrogen deficiency‐induced osteoporosis (Geng et al., 2019). However, given the transition from estrogen deficiency to aging and oxidative stress (Manolagas, 2010), and a recent study indicating that estrogen and aging‐inducing cellular senescence might play independent roles in the pathogenesis of osteoporosis (Farr et al., 2019), we sought in the current study to investigate the role and underlying mechanism of PQQ in the prevention of age‐related osteoporosis in mice.

Nuclear factor erythroid 2‐related factor 2 (Nrf2) is a broadly expressed transcription factor and a key regulator of antioxidant‐responsive genes that maintain cellular redox homeostasis, thus protecting against oxidative stress (Niture et al., 2014). Under basal conditions, Nrf2 is bound to its inhibitor, Kelch‐like ECH‐associated protein 1 (Keap1), which acts as an adapter for E3‐ubiquitin ligase and promotes the degradation of Nrf2 by the 26S‐proteasome (Cullinan et al., 2004); in contrast, under electrophilic/oxidative stress, the activity of Keap1 is inhibited, causing the release of Nrf2, which is translocated from the cytoplasm to the nucleus and binds to antioxidant response elements (AREs) in genes encoding antioxidant enzymes that protect cells against ROS (Suzuki & Yamamoto, 2015; Tonelli et al., 2018). Mounting evidence indicates that there is cross‐talk between Keap1‐Nrf2 and other proteins, such as p62, iASPP2, and MCM3, which can disrupt the normal Keap1‐Nrf2 signaling and facilitate Nrf2‐mediated cellular redox homeostasis (Ge et al., 2017; Komatsu et al., 2010; Mulvaney et al., 2016). The Nrf2 signaling pathway has also been shown to be protective in several diseases, including osteoporosis (Kim et al., 2014; Park et al., 2014; Sánchez‐de‐Diego et al., 2021; Sun et al., 2015; Yang, Zhang, et al., 2022), although the effect of Nrf2 activation on natural aging‐induced osteoporosis is unclear.

The purpose of this study was to investigate the role and mechanism of dietary PQQ supplementation in preventing natural age‐related osteoporosis. This study will provide insight into the mechanism whereby PQQ inhibits oxidative stress and prevents age‐related osteoporosis by indirectly inhibiting osteoclastic bone resorption via MCM3‐Keap1‐Nrf2 signaling activation in osteoblast‐lineage cells and provides evidence for PQQ as an attractive agent for the prevention and treatment of natural aging‐induced osteoporosis.

## 2 EXPERIMENTAL DETAILS

### 2.1 Animal experiments

A total of 24 six‐month‐old C57BL/6 male wild‐type mice were randomly divided into three groups. Young (6‐month‐old) and aging (18‐month‐old) wild‐type mice on a normal diet were used as the control groups, and the other two groups of mice were given a PQQ‐containing diet (4 mg/kg diet) from 12 months of age and 6 months of age, respectively. Nrf2^+/−^ mice were purchased from Cyagen Biosciences Inc. To determine whether Nrf2 is a key effector of PQQ, 6‐month‐old wild‐type and Nrf2^−/−^ mice were given the normal diet or the PQQ‐containing diet for 6 months. All animal experiments were performed in compliance with the guidelines approved by the Institutional Animal Care and Use Committee of Nanjing Medical University.

### 2.2 Microtomography (μ‐CT)

Vertebrae and femurs from indicated groups were removed and examined using μCT as previously described (Yang et al., 2020). Briefly, CT scanning was performed using the SkyScan1176 Micro‐CT system (Bruker) at a resolution of 9 μm for quantitative analysis. A region 1.8 mm wide and 2.0 mm long in trabecular bone of lumbar vertebrae (50 slices) was quantitatively analyzed for bone mineral density (BMD), trabecular bone volume per tissue volume (BV/TV), trabecular number (Tb.N), thickness (Tb.Th), and separation (Tb.Sp) using CTAn software. The region from 8.25 mm (500 slices) to 8.43 mm (520 slices) below the growth plate of the femurs was used for cortical thickness (Ct.Th) analysis using CTAn. The region from 10.05 mm (690 slices) to 10.5 mm (740 slices) below the growth plate of the femurs was analyzed for BMD, BV/TV, Tb.N, Tb.Th and Tb.Sp.

### 2.3 Histology and bone histomorphometry

At the time of euthanasia, lumbar vertebrae were fixed in PLP fixative buffer for 48 h. For routine histology analysis, decalcified bone using EDTA was dehydrated and embedded in paraffin, after which 5‐μm sections were prepared and bone sections were stained with the osteoclast marker tartrate‐resistant acid phosphatase (TRAP) and total collagen (T‐Col) as previously described (Yang et al., 2020). For assay of dynamic bone formation, mice were intraperitoneally injected with calcein (10 mg/kg, Sigma), 10 days after which they were given an injection of xylenol orange (XO) (90 mg/kg, Sigma). Dynamic bone formation was analyzed as previously described (Yang et al., 2020).

### 2.4 Cell cultures and treatment

Human and mouse BM‐MSCs were isolated and cultured as described previously (Yang, Li, et al., 2022). Primary osteoblasts from calvaria were isolated and cultured as described previously (Nollet et al., 2014). Briefly, calvaria were wished with PBS and cut into small fragments, which were then incubated in collagenase II and trypsin solution at 37°C in a shaking water bath. Bone pieces were then transferred into dishes in DMEM containing 10% FBS and 1% penicillin/streptomycin. Third‐passaged mouse osteoblasts were treated with PQQ for the indicated times.

### 2.5 Osteoclast formation assay

For osteoclast differentiation, mouse bone marrow monocytes (BMMs) isolated from femurs and tibias were cultured in DMEM supplemented with 10% FBS, 1% Penicillin/Streptomycin, and 50 ng/mL M‐CSF (PeproTech) for 3 days. Subsequently, adherent cells were further cultured in the presence of 50 ng/mL M‐CSF and 50 ng/mL RANKL with indicated treatment. For osteoblast and osteoclast co‐culture, osteoblasts isolated as described above were seeded into a 12‐well plate. BMMs were collected and cultured with osteoblasts in the presence of 1,25‐dihydroxyvitamin D3 (10 nM; Sigma) and PGE2 (1 μM; Sigma). The culture medium was changed every other day. Cells were fixed and stained for TRAP as previously described (Yang, Zhang, et al., 2022).

### 2.6 Immunohistochemistry staining

Immunohistochemistry staining was performed as previously described (Yang, Zhang, et al., 2022). Primary antibodies against rabbit anti‐Nrf2 (Proteintech, 16,396‐1‐AP), Mouse anti‐8‐OHdG (Abcam, ab62623), rabbit anti‐p16 (Abcam, ab211542), rabbit anti‐IL‐6 (Santa Cruz, sc‐1265), and rabbit anti‐IL‐1β (Abcam, ab9722) were used.

### 2.7 Prediction of potential targets of PQQ and molecular docking

The SDF (Structure Data File) file of PQQ was obtained from PubChem (https://pubchem.ncbi.nlm.nih.gov/compoundcompound/1024), and the effective targets were predicted using the PharmMapper Server (http://www.lilab‐ecust.cn/pharmmapper/) by employing the “All Targets” model. Autodock Vina 1.1.2 was employed to investigate the binding affinity and binding sites between PQQ and MCM. Briefly, the SDF file of PQQ was converted into PDBQT format by using AutoDock tools. The 3D crystal structure of the MCM3 was obtained from the Uniprot (http://www.uniprot.org, PDB ID: AF‐P25206‐F1). The docking protocol was generated as described previously (Li et al., 2020), and the partial diagram of molecular docking was generated using the PyMol software.

### 2.8 Plasmid construction

Plasmids, including FLAG‐Keap1, HA‐Nrf2, and His‐MCM3, were purchased from PPL (Public Protein/Plasmid Library). The indicated shRNAs targeting MCM3 and Fbn1 were cloned to the lentivirus shRNA vector as previously described (Chen et al., 2018). For ARE‐driven luciferase reporter assay, a total of 8 ARE copies was cloned to a pGL3‐promoter as we previously described (Yang, Zhang, et al., 2022). All constructs were verified by DNA sequencing (Tsingke).

### 2.9 Lentivirus production

Lentivirus particles were generated from HEK293T cells as previously described (Yang, Zhang, et al., 2022). Briefly, HEK293T cells were co‐transfected with transfer plasmid and packaging plasmids by Lipofectamine 2000 transfection reagent (Invitrogen). Viral particle‐containing supernatants were harvested 24 and 48 h later and were concentrated by ultra‐centrifugation (4000 × g for 3 h).

### 2.10 Co‐immunoprecipitation

For exogenous and endogenous co‐immunoprecipitation (Co‐IP), cells were lysed in IP lysis buffer (20 mM Tris [pH 7.8], 150 mM NaCl, 0.2% NP‐40, 10% glycerol, supplemented with protease inhibitors cocktail [Roche Complete]) for 15 min, after which the cleared lysates were collected by centrifugation and incubated with the indicated antibodies or control IgGs coupled to Protein A/G agarose beads (Invitrogen) at 4°C overnight under rotation. After being washed three times with IP lysis buffer, the immunoprecipitants were eluted by boiling in Laemmli loading buffer and analyzed using immunoblotting.

### 2.11 Detection of ROS

ROS evaluation of living cells in vitro using Dihydroethidium (DHE, ApexBio) was performed as previously described (Yang, Zhang, et al., 2022). For the detection of ROS in vivo, bone was sectioned without fixing using a transparent film, and sections were incubated in DHE at 37°C for 30 mins. The fluorescence was captured under a microscope, and the intracellular intensity of DHE was quantified using Image J.

### 2.12 ChIP‐qPCR

ChIP‐qPCR was performed using the ChIP kit (Millipore) as previously described (Yang et al., 2020). The antibodies used in ChIP‐qPCR were as follows: rabbit anti‐IgG (Abcam, ab172730) and rabbit anti‐Nrf2 (Proteintech). The enriched DNA was used for qPCR to detect the putative ARE of Fbn1. The sequences of primers were listed in Table S1.

### 2.13 Dual luciferase assay

The indicated length of the Fbn1 promoter and its mutant were directly synthesized and cloned to pGL3‐basic (Promega). For ARE‐driven luciferase reporter assay, BM‐MSCs were co‐transfected with ARE‐driven luciferase, pcDNA3.1‐Nrf2, and 0.05 μg of renilla followed without or with PQQ treatment. Forty‐eight hours later, relative luciferase activity was measured using a kit (Promega) according to the manufacturer's instructions. The promoter sequences of Fbn1 and its mutant sequence cloned to pGL4.17 are listed in Table S3.

### 2.14 Western blot

Whole‐cell lysates extraction and immunoblotting were performed as previously described (Yang et al., 2020). Primary antibodies against rabbit anti‐Nrf2 (Proteintech, 16,396‐1‐AP), rabbit anti‐p21 (Abcam, ab188224), mouse anti‐p16 (Santa Cruz, sc‐1661), rabbit anti‐Rankl (Proteintech, 23,408‐1‐AP), rabbit anti‐HO‐1 (Proteintech, 10,701‐1‐AP), rabbit anti‐Keap1 (Proteintech, 10,503‐2‐AP), rabbit anti‐ubiquitin (Cell Signaling Technology, 43,124), rabbit anti‐MCM3 (Proteintech, 15,597‐1‐AP), mouse anti‐Flag (Sigma, F1804), mouse anti‐His (Proteintech, 66,005‐1‐Ig), mouse anti‐GAPDH (Beyotime, AF0006), and mouse anti‐β‐Actin (Beyotime, AF2811) were used in this study. GAPDH or β‐Actin was used to normalize changes in specific gene expressions detected using Western blots.

### 2.15 RNA isolation and real‐time RT‐PCR

Total RNA extraction, cDNA synthesis, and real‐time RT‐PCR were performed as previously described (Yang, Zhang, et al., 2022). Gapdh was amplified at the same time to normalize gene expression. The PCR primer sequences used in this study are shown in Table S2.

### 2.16 Statistical analysis

Measured data were described as mean ± SD. The statistical analyses were performed using GraphPad Prism (Version 8.0). Two‐tailed Student's *t*‐test was used to compare differences between groups. For multiple comparisons, one‐way or two‐way ANOVA analysis of variance was used. *p* values <0.05, <0.01, and <0.001 were considered statistically significant (*, **, ***).

## 3 RESULTS

### 3.1 PQQ supplementation prevents natural aging‐related bone loss and skeletal aging in mice

**FIGURE 1:**
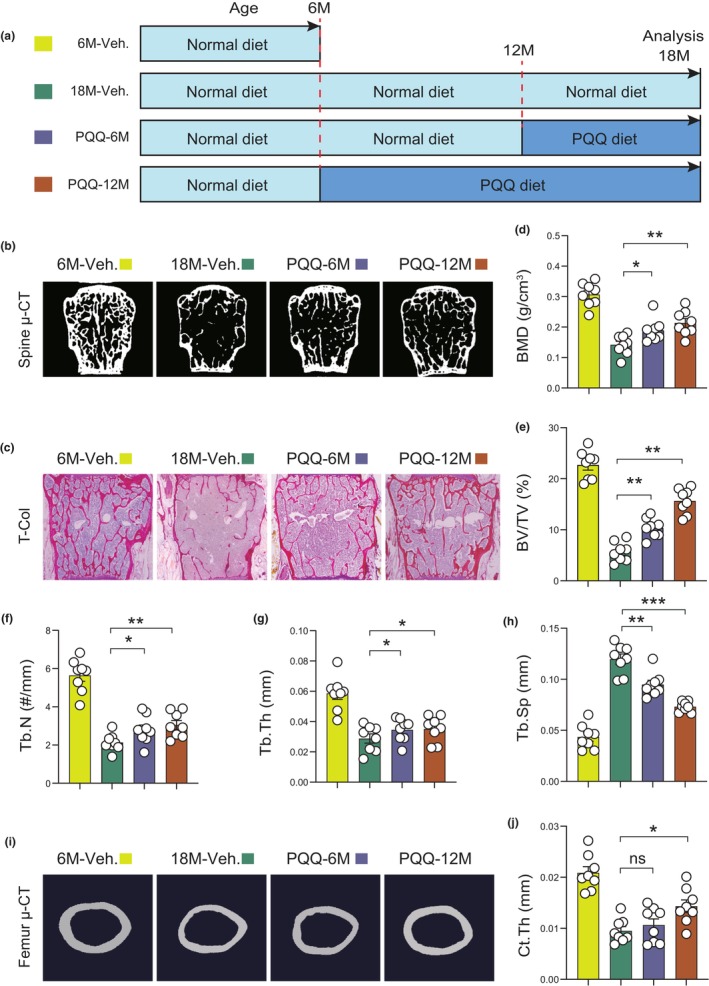
PQQ supplementation alleviates the bone aging phenotype in aging wild‐type mice. (a) Experimental design for investigating the effects of PQQ on natural aging‐induced bone loss; wild‐type mice at the age of 6 and 12 months were given the PQQ diet (4 mg/kg standard feed) for 12 and 6 months, respectively. Control mice were given the normal diet. Mice were sacrificed and skeletal phenotypes analyzed at indicated ages. (b) Representative μCT scans and (c) Total collagen staining of lumbar vertebrae from indicated groups of mice. Microtomography indices were measured as (d) BMD, (e) BV/TV, (f) Tb.N, (g) Tb.Th, and (h) Tb.Sp. (i) Representative μCT images of bone microarchitecture at the femur diaphysis of indicated groups of mice. (j) Quantification of μCT‐derived cortical thickness (Ct.Th). One‐way ANOVA. *p < 0.05, **p < 0.01, ***p < 0.001. ns, not significant.

To assess whether PQQ supplementation can prevent natural aging‐induced osteoporosis, wild‐type male mice were given a PQQ‐containing diet (4 mg PQQ/kg diet) from the age of 6 and 12 months, respectively, and the other two groups (6‐ and 18‐month‐old male mice) were given a normal diet as the control groups. All mice were sacrificed, and skeletal phenotypes were analyzed using micro‐CT and total collagen (T‐Col) staining (Figure 1a–c). Compared with age‐matched littermates given the normal diet, natural aging‐related bone loss of vertebrae and femurs were significantly alleviated upon dietary supplementation of PQQ for 6 or 12 months, as determined by the increase of bone mineral density (BMD), trabecular bone volume (BV/TV), trabecular number (Tb.N), trabecular thickness (Tb.Th), and the decrease of trabecular separation (Tb.Sp) (Figure 1d–h; Figure S1a–g). Femur cortical thickness was significantly increased in aged mice following PQQ treatment for 12 months as compared to controls (Figure 1i,j). To further confirm our hypothesis, we also administered PQQ to 12‐month‐old wild‐type mice for 12 months, analyzed bone phenotypes at the age of 24 months and results showed that aging‐related bone loss of vertebrae was also significantly alleviated upon PQQ treatment as determined by BMD, BV/TV, Tb.N, Tb.Th, and Tb.Sp (Figure S2a–f). Interestingly, natural aging‐induced bone loss phenotypes were much more substantially prevented when PQQ was supplemented at a younger age i.e., starting from 6 months of age than if PQQ was initiated at 12 months of age (Figure 1b–j; Figure S1a–g). Of note, dietary supplements containing PQQ have been found to be safe in humans and approved by the U.S. Food and Drug Administration (FDA) (Akagawa et al., 2016; Office of Food Additive Safety (FHS‐200) Center for Food Safety and Applied Nutrition Food and Drug Administration; Washinton, 2017), and indeed, our results showed that total body weight, liver damage markers (e.g., aspartate aminotransferase [AST] and alanine aminotransferase [ALT]), and kidney damage markers (e.g., serum creatinine) were not significantly altered in PQQ‐supplemented aged mice relative to the control (Figure S3a–d). These results indicated that PQQ supplementation can prevent natural aging‐related osteoporosis in wild‐type mice, making it an ideal option for alleviating natural aging‐induced disease osteoporosis in humans if the same results can be demonstrated in aging humans.

To assess the mechanism whereby PQQ supplementation can rescue the bone aging phenotype, we examined the changes of oxidative stress, DNA damage, cellular senescence, and SASP in the indicated groups of mice mentioned in Figure 1a. Results revealed that the percentage of Nrf2‐positive osteocytes and the mRNA levels of heme oxygenase 1 (HO1), SOD2, GST, and glutathione peroxidase 7 (GPx7) were significantly increased and the percentage of 8‐OHdG‐positive osteocytes in bone tissues were significantly decreased in PQQ‐supplemented mice as compared to vehicle‐treated mice (Figure S4a–d,h). Cellular senescence and SASP‐related parameters, including the percentage of p16‐, IL‐6‐ and IL‐1β‐positive osteocytes (Figure S4e–g,i–k), the expression levels of p16 and p21 in bone tissues (Figure S4m,n), and the mRNA expression levels of *p16, Mmp3, IL‐6*, and *IL‐1β* in bone tissues (Figure S4l) were significantly decreased in PQQ‐supplemented mice as compared to vehicle‐treated mice. These results suggested that PQQ can rescue the natural bone aging phenotype by inhibiting oxidative stress and cellular senescence.

### 3.2 PQQ rescues aging‐induced reduction of osteoblastic bone formation and augmentation of osteoclastic bone resorption by inhibiting oxidative stress

**FIGURE 2:**
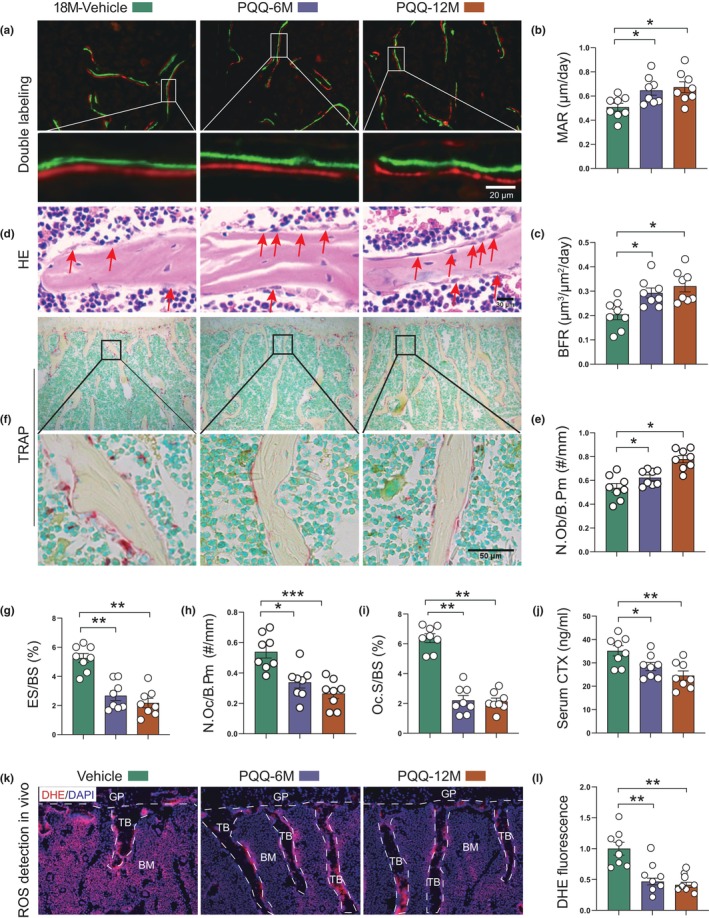
PQQ rescued aging‐induced ROS accumulation and increased bone resorption. (a) Representative images of dual calcein‐xylenol orange (XO) labeling (DL) of lumbar vertebrae. (b) Quantification of mineral apposition rate (MAR) and (c) bone formation rate normalized to bone surface (BFR/BS). (d) Representative HE staining images of vertebral trabecular sections and (e) the quantitative analysis of the number of osteoblasts per bone perimeter (N.Ob/B.Pm). (f) Representative sections stained histochemically for TRAP and the quantitative analysis of (g) eroded surface normalized to bone surface (ES/BS), (h) osteoclast number (N.Oc/B.Pm, #/mm) and (i) osteoclast surface per bone surface (Oc.S/BS). (j) Serum CTx levels (ng/mL). (k) ROS levels in the lumbar vertebrae determined using dihydroethidium (DHE) staining in vivo. (l) Quantitative analysis of DHE fluorescence fold‐change of (j). One‐way ANOVA. *p < 0.05, **p < 0.01. ns, not significant.

We next sought to determine the relative contributions of osteoblast and osteoclast activity to the alleviation of bone loss in PQQ‐supplemented aged mice. We firstly performed histomorphometric analysis to evaluate static and dynamic parameters of bone formation. Results showed that bone mineral apposition rate (MAR) and bone formation rate (BFR) were significantly elevated in PQQ‐supplemented aging mice (Figure 2a–c). Consistently, the number of osteoblasts per bone perimeter (N.Ob/B.Pm) based on HE staining (Figure 2d,e) was significantly increased in PQQ‐supplemented mice compared to vehicle‐treated mice. Interestingly, there was significantly decreased bone resorption in PQQ‐supplemented mice as compared to vehicle‐supplemented controls, as determined by decreased eroded surface normalized to bone surface (ES/BS) (Figure 2g), osteoclast number (N.Oc/B.Pm) (Figure 2h), osteoclast surface per bone surface (Oc.S/BS) (Figure 2i), and serum CTX‐I level (Figure 2j) in PQQ‐supplemented mice compared to vehicle‐treated mice. Tartrate‐resistant acid phosphatase (TRAP) staining confirmed the decrease of osteoclasts along the surface of trabecular bone in PQQ‐supplemented mice (Figure 2f). Moreover, DHE staining of vertebral bone tissues revealed that PQQ significantly reduced ROS levels in bone tissues in vivo (Figure 2k,l). These results suggested that PQQ alleviates natural aging‐induced osteoporosis by increasing bone formation and inhibiting osteoclastic bone resorption through decreasing oxidative stress.

### 3.3 PQQ increases stress response capacity and reduces Rankl secretion of osteoblasts from aged mice by activating Nrf2‐ARE signaling

**FIGURE 3:**
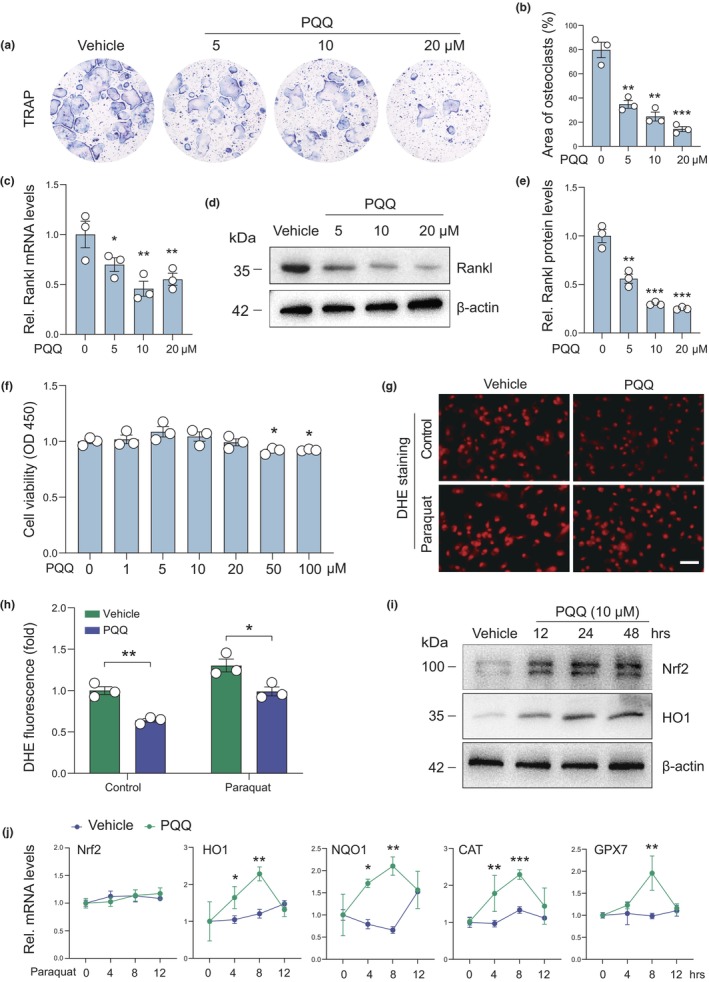
PQQ increases stress response and decreases Rankl production in osteoblasts from aged mice by activating Nrf2‐ARE signaling. (a, b) Osteoclastogenesis by OB‐OC co‐culture in vitro using osteoblasts from 18‐month‐old wild‐type mice treated with or without PQQ for 3 days: (a) TRAP staining in the co‐culture system and (b) the quantification of the area of TRAP^+^ multinucleated cells (MNCs, nuclei ≥3). (c–e) Rankl expression in vitro using osteoblasts from 18‐month‐old wild‐type mice treated with or without PQQ for 48 h: (c) Real‐time PCR detection of Rankl; (d) Western blot detection and (e) quantification of Rankl protein levels. (f) The effect of PQQ on aged osteoblasts viability was determined using CCK8 assay. Two‐tailed Student's *t*‐test. *p < 0.05, **p < 0.01, ***p < 0.001. (g) ROS levels in paraquat‐treated aged osteoblasts in the presence or absence of PQQ were detected using DHE staining. (h) Quantitative analysis of (g). Two‐way ANOVA. *p < 0.05, **p < 0.01. (i) Western blot detection of Nrf2 and HO1 protein levels in paraquat‐treated aged osteoblasts in the presence or absence of PQQ at indicated times. (j) Real‐time PCR detection of Nrf2, HO1, Nqo1, CAT, and GPX7 mRNA levels in the presence or absence of PQQ in paraquat‐treated aged osteoblasts at indicated times. Two‐way ANOVA. *p < 0.05, **p < 0.01.

Next, we sought to investigate in greater detail the mechanism whereby PQQ inhibits osteoclast activity. BMMs isolated from 16‐month‐old wild‐type mice were induced to differentiate into osteoclasts by adding Rankl and M‐CSF with or without PQQ. Additionally, we induced osteoclasts by OB‐OC co‐culture. Osteoblasts isolated from long bones of 16‐month‐old wild‐type mice were treated with PQQ for 3 days, following which they were co‐cultured with BMMs in the presence of 1,25(OH)_2_D_3_ and Prostaglandin E2 (PGE2). Interestingly, we found that direct formation of osteoclasts from BMMs was not significantly changed in the presence of PQQ (Figure S5a,b), whereas osteoclast formation of BMMs in the co‐culture system was significantly decreased by treating osteoblasts with PQQ (Figure 3a,b). Furthermore, the mRNA and protein expression levels of Rankl were significantly reduced in PQQ‐treated osteoblasts compared to vehicle‐treated control (Figure 3c–e). Osteoblast viability was not significantly altered upon PQQ treatment for 24 h at concentrations of 1, 5, 10, and 20 μM (Figure 3f), indicating that the decline of Rankl in PQQ‐treated osteoblasts was not caused by decreased cell viability. Increased Rankl production was reported to be associated with increased oxidative stress in osteoblasts (Baek et al., 2010; Choi, 2012; Nollet et al., 2014; Rana et al., 2012). Senescent cells appear to have decreased capacity to initiate antioxidant signaling in the presence of stress stimulation, in association with decreased expression of Nrf2, the master regulator of oxidative stress (Meng et al., 2017). Consistent with this observation, we found here that senescent osteoblasts exhibited decreased Nrf2 expression and stress response capacity following treatment with paraquat (an oxidative stress inducer), as well as increased oxidative stress and Rankl production (Figure S6a–i). Furthermore, Nrf2‐deficient osteoblasts resulted in higher Rankl production (Figure S7a,b) and increased osteoclast formation (Figure S7c,d) in the co‐culture system. Moreover, DHE staining showed that ROS levels in osteoblasts were significantly reduced in PQQ‐treated osteoblasts relative to vehicle‐treated controls in the absence or presence of paraquat (Figure 3g,h). Furthermore, the protein, but not mRNA expression levels of Nrf2 were significantly increased in PQQ‐treated osteoblasts relative to vehicle‐treated control osteoblasts (Figure 3i,j). In addition, in the presence of paraquat, the mRNA levels of antioxidant‐related genes downstream of Nrf2 were not significantly changed in vehicle‐treated senescent osteoblasts; however, these genes were significantly up‐regulated in PQQ‐treated senescent BM‐MSCs (Figure 3i,j). These results suggested that PQQ increases stress response capacity and inhibits Rankl production of osteoblasts, thus directly reducing osteoclastogenesis via activating Nrf2‐ARE signaling.

### 3.4 PQQ binds to and promotes protein stability of MCM3, competing with Nrf2 for binding to Keap1

**FIGURE 4:**
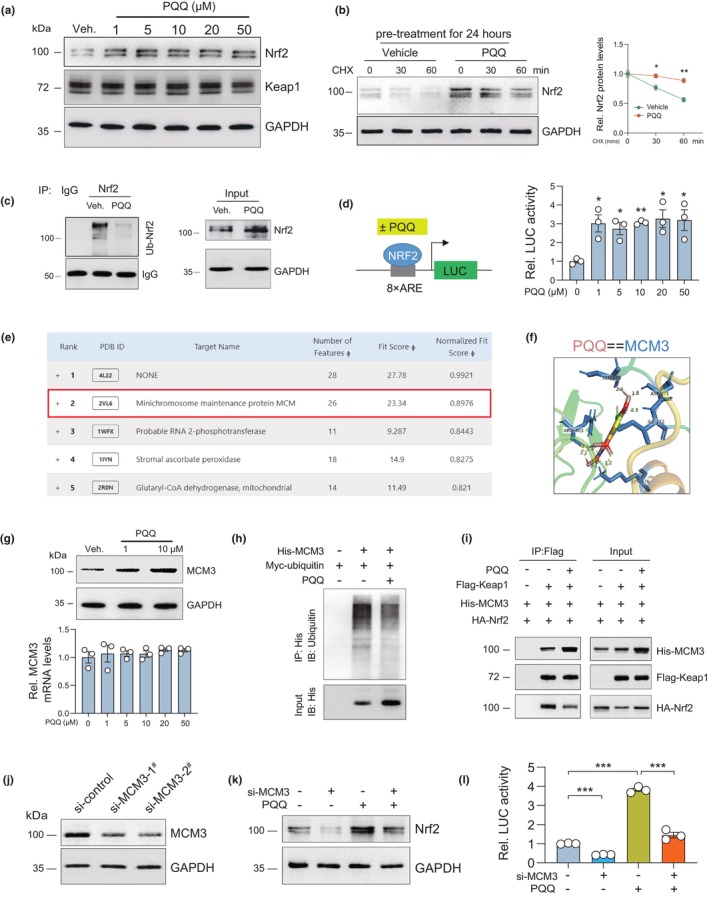
PQQ binds to and promotes protein stability of MCM3, competing with Nrf2 for binding to Keap1. (a) Western blot detection of Nrf2 and Keap1 in human BM‐MSCs treated with vehicle or indicated dose of PQQ. (b) Western blot detection and quantification of Nrf2 protein levels in hBM‐MSCs pretreated with vehicle or PQQ for 24 hours, following which cells were treated with CHX for indicated times. Two‐way ANOVA. *p < 0.05, **p < 0.01. (c) Ubiquitin‐Nrf2 (Ub‐Nrf2) was determined by immunoprecipitation (IP) in hBM‐MSCs treated with vehicle or PQQ for 24 h. (d) Relative luciferase activity driven by ARE in hBM‐MSCs following vehicle or PQQ treatment for 24 h was determined using dual luciferase assay. Two‐tailed Student's *t*‐test. *p < 0.05, **p < 0.01. (e) The top 5 potential targets of PQQ ranked by the normalized fit score in descending order. (f) Binding mode between PQQ and MCM3. (g) Western blot and qPCR detection of MCM3 levels in hBM‐MSCs treated with vehicle or PQQ for 24 h. (h) Ubiquitin‐MCM3 (Ub‐MCM3) was determined by immunoprecipitation (IP) in hBM‐MSCs treated with vehicle or PQQ for 12 h. (i) Exogenous interactions between Flag‐Keap1 and His‐MCM3/HA‐Nrf2 in 293T treated with or without PQQ were determined using co‐immunoprecipitation (Co‐IP) and Western blot. (j) Knock‐down efficiency of si‐MCM3. (k) Western blot detection of Nrf2 in hBM‐MSCs treated with vehicle or PQQ following MCM3 knockdown. (l) Relative luciferase activity driven by ARE in hBM‐MSCs treated with vehicle or PQQ following MCM3 knockdown. Two‐way ANOVA. **p < 0.01.

To investigate the mechanism underlying how PQQ activates Nrf2‐ARE signaling, human BM‐MSCs isolated from aged osteoporosis patients were treated with indicated concentrations of PQQ for 24 h, and the expression levels of Nrf2 and Keap1 (the main regulator of Nrf2) were detected using Western blot and real‐time PCR. Results showed that the protein expression level of Nrf2, but not its mRNA expression level was significantly increased in PQQ‐treated hBM‐MSCs relative to vehicle‐treated control (Figure 4a; Figure S8a), indicating that PQQ might regulate Nrf2 at a post‐translational level. Indeed, further results showed that Nrf2 protein degradation was delayed (Figure 4b), and the expression level of ubiquitin‐Nrf2 was significantly decreased in PQQ‐pretreated hBM‐MSCs as compared with vehicle‐pretreated control (Figure 4c). In addition, ARE‐driven luciferase activity was significantly increased in hBM‐MSCs treated with PQQ for indicated times (Figure 4d), indicating that the transcriptional activity of Nrf2 was increased upon PQQ treatment. Keap1 is the master regulator of Nrf2 at the post‐translational level; however, neither the protein nor the mRNA expression of Keap1 was increased in PQQ‐treated hBM‐MSCs relative to vehicle‐treated control (Figure 4a; Figure S8a). To determine the direct target of PQQ, the structural data file of PQQ was submitted to the Pharmmapper, selecting the “All Targets” model, and the top 5 potential targets ranked by the normalized fit score in descending order were listed (job ID: 220330013617) (Figure 4e). The detailed results were uploaded as a separate file (Table S4). Among these candidates, MCM were most likely to be the target of PQQ based on the normalized fit score (Figure 4e). Furthermore, the AutoDock Vina binding test results revealed that PQQ might favorably bind to MCM3 (minichromosome maintenance complex component 3) relative to other isoforms of MCM based on the binding energy and hydrogen bonds formed (Figure 4f; Figure S9a,b). Keap1 is considered as the main regulatory effector of Nrf2, and it has been reported that MCM3 can compete with Nrf2 for binding to Keap1, thus inhibiting Nrf2 degradation (Mulvaney et al., 2016; Tamberg et al., 2018). To test the effect of PQQ‐MCM3 binding on MCM3 expression and PQQ‐induced Nrf2 signaling activation, we examined the expression of MCM3 and the Keap1‐Nrf2 interaction using Western blots and co‐immunoprecipitation. We found that the protein levels of MCM3 were significantly decreased in aging bones (3 vs. 18 M) and in passage‐induced replicative senescent (P4 vs. P14) hBM‐MSCs (Figure S9c–f). In addition, the protein, but not mRNA expression level of MCM3 was significantly higher in PQQ‐treated human BM‐MSCs than in vehicle‐treated control (Figure 4g). Protein degradation experiments showed that the ubiquitination and protein degradation rate of MCM3 were significantly inhibited in PQQ‐treated hBM‐MSCs relative to vehicle‐treated controls (Figure 4h; Figure S8b). Furthermore, co‐immunoprecipitation results showed that PQQ significantly increased the interaction between Keap1 and MCM3 and decreased the interaction between Keap1 and Nrf2. (Figure 4i). Moreover, the reduced interaction between Nrf2 and Keap1 and the transcriptional activity of Nrf2 induced by PQQ were significantly blocked upon MCM3 knockdown in hBM‐MSCs (Figure 4j–l). These results indicated that PQQ could maintain the expression levels of Nrf2 in repeatedly passaged BM‐MSCs by directly binding to MCM3 to augment its protein stability; MCM3 thus competes with Nrf2 for binding to Keap1 and reduces the degradation of Nrf2 by the ubiquitin system.

### 3.5 Nrf2 activation partly inhibits osteoclast differentiation by upregulating Fbn1 in BM‐MSCs

**FIGURE 5:**
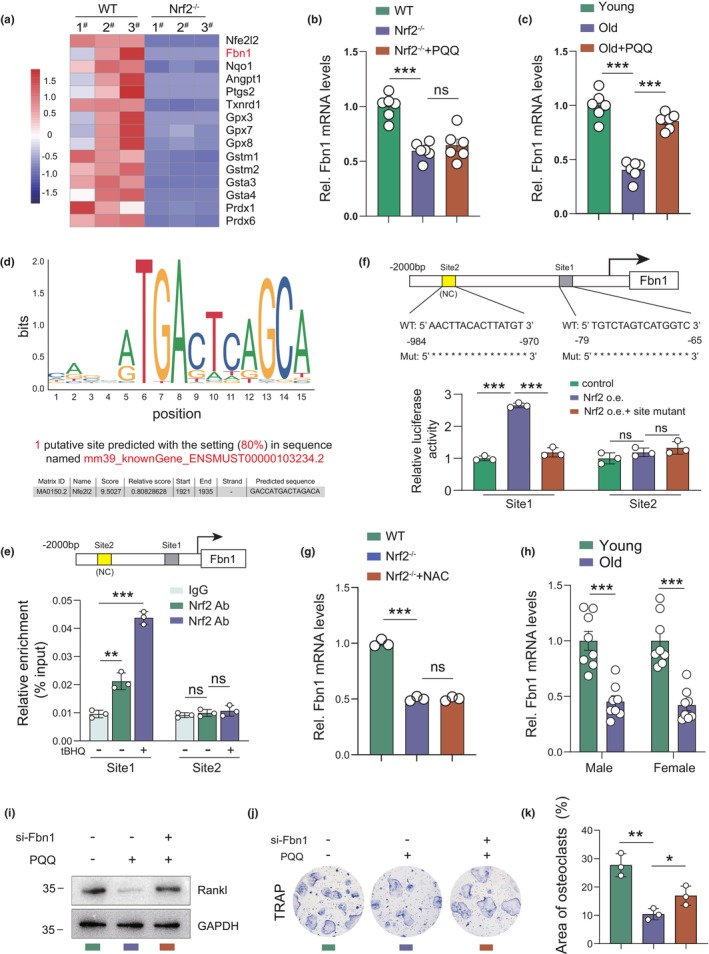
Nrf2 decreased osteoclast differentiation by enhancing Fbn1 transcription in BM‐MSCs. (a) RNA‐seq heatmap showing the mentioned genes expressed in BM‐MSCs isolated from WT and Nrf2^−/−^ mice. (b) The mRNA levels of Fbn1 in BM‐MSCs from WT, Nrf2^−/−^ mice and PQQ‐treated Nrf2^−/−^ mice. (c) The mRNA levels of Fbn1 in BM‐MSCs from young (3‐month‐old), old (18‐month‐old), and PQQ‐treated 18‐month‐old WT mice. (d) A predictive Nrf2‐binding element in mouse Fbn1 promoter region. (e) Chromatin immunoprecipitation (ChIP) with Nrf2 antibody or IgG antibody were performed in vehicle‐ or tBHQ‐treated mouse BM‐MSCs, and relative enrichment of Fbn1 promoter was determined using qPCR assay. (f) Mouse Fbn1 promoter or Fbn1 promoter mutant Luc‐plasmids were transfected into mouse BM‐MSCs following lentivirus‐mediated Nrf2 overexpression, and relative luciferase activity was analyzed after 48 h. (g) The mRNA levels of Fbn1 in BM‐MSCs from WT, Nrf2^−/−^ mice and NAC‐treated Nrf2^−/−^ mice. (h) The mRNA levels of Fbn1 in bones from young (3‐month‐old) and old (18‐month‐old) female and male wild‐type mice. (i) Western blot detection of Rankl in vehicle‐ or PQQ‐treated mouse osteoblasts in the presence or absence of si‐Fbn1. (j) TRAP staining in the co‐culture system in indicated groups. (k) Quantitative analysis of the area of osteoclasts in (j). Two‐tailed Student's *t*‐test. *p < 0.05, **p < 0.01, ***p < 0.001. ns, not significant.

To examine whether PQQ‐mediated Nrf2 activation can directly inhibit osteoclast differentiation in the OB‐OC co‐culture system independent of stress response regulation, RNA‐seq was performed on primary BM‐MSCs isolated from WT and Nrf2^−/−^ mice. The results showed that aside from antioxidant‐related genes, fibrillin‐1 (Fbn1) was significantly decreased in Nrf2^−/−^ BM‐MSCs as compared to wild‐type controls (Figure 5a). Fbn1 deficiency has been reported to result in bone loss with constitutively enhanced bone resorption in mice (Smaldone et al., 2016), and Fbn1‐deficient osteoblasts have been shown to stimulate pre‐osteoclast differentiation more than wild‐type osteoblasts (Tiedemann et al., 2013). Real‐time qPCR results showed that PQQ significantly increased Fbn1 expression in aged wild‐type BM‐MSCs, but not in age‐matched Nrf2^−/−^ BM‐MSCs, indicating that the increase of Fbn1 induced by PQQ depends on Nrf2 (Figure 5b,c). Based on these results, we speculated that PQQ might regulate the transcription of Fbn1 through Nrf2. A predicted ARE was detected in the promoter of Fbn1 (Figure 5d), and the chromatin immunoprecipitation (ChIP) assay was used to confirm its involvement. Thus, ChIP results showed that Nrf2 bound to the promoter region of Fbn1 under physiological conditions; this binding was significantly increased by treatment with tert‐butylhydroquinone (tBHQ, a classic Nrf2 activator) (Figure 5e). Dual luciferase assays showed that luciferase activity, driven by a Fbn1 promoter containing the predicted ARE, was significantly increased following Nrf2 overexpression, while this effect was abolished when the predicted ARE was mutated (Figure 5f). Furthermore, the antioxidant reagent NAC did not rescue the decreased Fbn1 mRNA level in Nrf2^−/−^ BM‐MSCs (Figure 5g), indicating that the increased Fbn1 induced by Nrf2 activation was independent of decreased oxidative stress. Meanwhile, we detected Fbn1 expression in mouse bone tissues and found that the mRNA expression level of Fbn1 was also significantly down‐regulated in aging bone tissues relative to young controls (Figure 5h). Moreover, co‐culture experiments and TRAP staining results showed that Fbn1 knockdown in mouse calvarial osteoblasts significantly increased Rankl production in osteoblasts, and enhanced osteoclast differentiation of BMMs (Figure S10a–d). Notably, Fbn1 knockdown in osteoblasts significantly blocked PQQ‐induced reduction of Rankl production in osteoblasts and osteoclast differentiation of BMMs (Figure 5i–k). These results demonstrated that Nrf2 activation can inhibit osteoclast differentiation by transcriptionally upregulating Fbn1 in osteoblast‐lineage cells.

### 3.6 Nrf2 depletion largely blocks the inhibitory effects of PQQ on oxidative stress, osteoclast activity, and aging‐related osteoporosis

**FIGURE 6:**
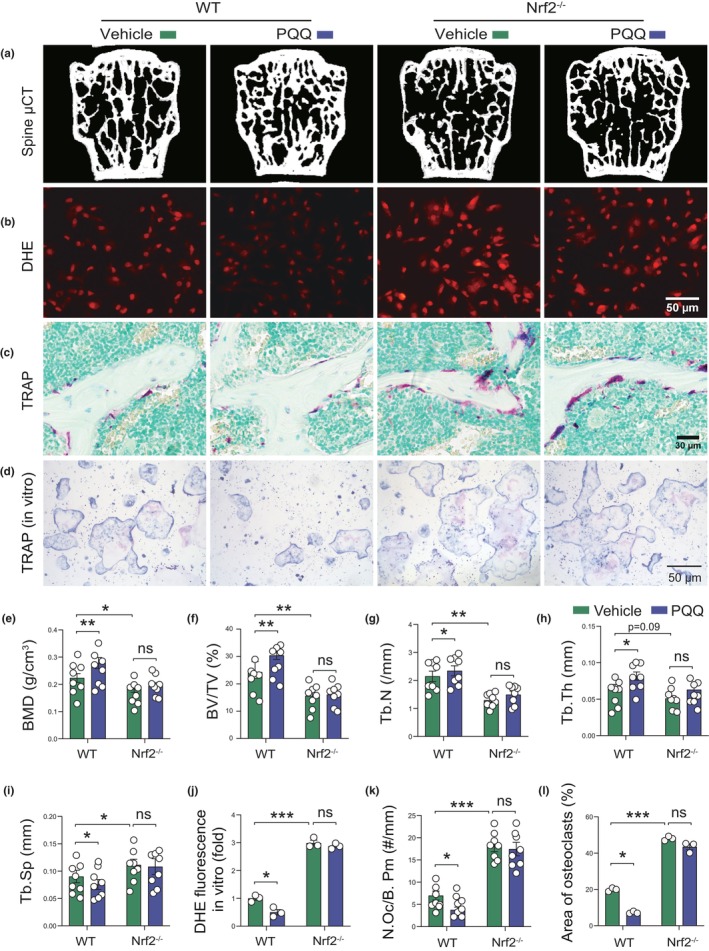
Nrf2 knockout blunts the preventing effects of PQQ on aging‐related osteoporosis, oxidative stress, and osteoclastic bone resorption. (a) Representative μCT scans of 3D longitudinal reconstructions. Microtomography indices were measured as (e) BMD, (f) BV/TV, (g) Tb.N, (h) Tb.Th and (i) Tb.Sp. (b) ROS levels in WT or Nrf2^−/−^ osteoblasts in the presence or absence of PQQ were detected using DHE staining. (j) DHE fluorescence analysis of (b). (c) Representative images stained histochemically for TRAP and (k) the quantitation of the number of osteoclasts per bone perimeter (N.Oc/B.Pm, #/mm). (d) WT and Nrf2^−/−^ osteoblasts were treated with vehicle or PQQ for 3 days, after which they were co‐cultured with BMMs for osteoclast differentiation, and osteoclasts were detected using TRAP staining. (l) Quantitative analysis of the area of TRAP‐positive multinucleated cells. Two‐way ANOVA. *p < 0.05, **p < 0.01. ns, not significant.

In order to determine whether Nrf2 is the key mediator of the inhibitory effects of PQQ on oxidative stress, osteoclast activity, and aging‐related osteoporosis, 6‐month‐old wild‐type and Nrf2^−/−^ male mice were fed with a PQQ‐ or vehicle‐containing diet for 6 months, and skeletal phenotypes of these mice were analyzed using μCT and histomorphometry at the age of 12 months. We firstly found that Nrf2^−/−^ mice at the age of 18 months exhibited aging‐related bone loss phenotypes, including the reduction of BMD, bone volume, trabecular number and thickness and the increase of trabecular separation (Figure S11a–f), as well as bone aging phenotypes, including the increase of DNA damage, osteocyte senescence, and SASP (Figure S11g–p). Furthermore, results showed that BMD, trabecular number, volume, and thickness were significantly elevated in PQQ‐supplemented wild‐type mice, but not in PQQ‐supplemented Nrf2^−/−^ mice (Figure 6a,e–h), whereas trabecular separation, oxidative stress in osteoblasts, osteoclast activity in vivo, and osteoclastogenesis in co‐culture system were significantly reduced in PQQ‐supplemented wild‐type mice, but not in PQQ‐supplemented Nrf2^−/−^ mice (Figure 6b–d,i–l). These results indicated that Nrf2 is essential to mediate the inhibitory effects of PQQ on oxidative stress, osteoclast activity, and aging‐related osteoporosis.

## 4 DISCUSSION

Estrogen and aging‐inducing cellular senescence have been reported to play independent roles in the pathogenesis of osteoporosis (Farr et al., 2019). We have previously reported that PQQ can prevent estrogen deficiency‐induced osteoporosis (Geng et al., 2019); however, whether PQQ can alleviate aging‐related osteoporosis and the specific mechanism remains unclear. Here, we showed that dietary PQQ supplementation could not only promote bone formation of osteoblasts, but also inhibit bone resorption through the activation of stress response and the upregulation of Fbn1 mediated by MCM3‐Keap1‐Nrf2, thus preventing natural aging‐related osteoporosis.

PQQ was identified as a redox cofactor with strong radical‐scavenging activity, and it has been reported to alleviate several disorders (Jonscher et al., 2017). Our previous studies have demonstrated that PQQ can prevent oophorectomy‐induced osteoporosis (Geng et al., 2019). Aging has been recognized as the most important inducer of osteoporosis in both males and females. Here, we administered PQQ to wild‐type mice from 6 or 12 months of age for 12 or 6 months, respectively, and found that PQQ alleviated natural aging‐induced osteoporosis. Of note, PQQ has served as a safe food supplement in some countries, making it an ideal option for the prevention of aging‐related diseases, including osteoporosis. Indeed, we found here that PQQ treatment did not alter liver and kidney damage‐related markers. Overall, our findings further confirmed that PQQ could be a potential drug beneficial for the prevention and treatment of aging‐related osteoporosis. Interestingly, this skeletal protective effect of PQQ appears to be more pronounced when PQQ is provided at a relatively younger age. This suggests that PQQ needs to be assessed in humans in future studies and might be useful in delaying the onset of osteoporosis by increasing peak bone mass in youth.

In our studies, PQQ alleviated natural aging‐related osteoporosis not only by stimulating osteoblastic bone formation, but also by inhibiting osteoclastic bone resorption. Previous studies have demonstrated that upregulation of Nrf2 in osteoblasts can indirectly reduce osteoclast formation by inhibiting the secretion of Rankl in osteoblasts (Narimiya et al., 2019; Rana et al., 2012). Increased Rankl production has been reported to be associated with increased oxidative stress in osteoblasts (Baek et al., 2010; Choi, 2012; Nollet et al., 2014; Rana et al., 2012). PQQ also significantly increased Nrf2 expression and decreased osteoclastic bone resorption in aging mice. It has been reported that the stress response capacity declined in senescent cells (Meng et al., 2017), and we found that PQQ could partially restore the oxidative stress response capacity of repeatedly passaged BM‐MSCs by upregulating Nrf2 expression, while inhibiting osteoclast formation by decreasing Rankl secretion. Overall, our results suggest that PQQ can increase the protein level of Nrf2 to enhance the oxidative stress response and decrease Rankl production in osteoblasts, thus inhibiting osteoclast activity. Interestingly, here we did not observe a direct inhibitory effect of PQQ on osteoclast differentiation of BMMs, which is inconsistent with a previous study (Kong et al., 2013). This may be caused by the different sources of BMMs used, with the previous study using BMMs from young rats and this study using BMMs from aging mice.

In our study, PQQ treatment not only decreased oxidative stress and inhibited osteoclast activity in vivo in aging mice, but also increased osteoblastic bone formation. This appears to be consistent with the crucial role for Nrf2‐antioxidant signaling for redox homeostasis and osteoblast survival. Indeed, a recent study reported that Nrf2 protects osteoblasts from cell death caused by glucocorticoid‐induced oxidative stress and promotes osteogenesis (Rai et al., 2022). In addition, we here found that PQQ‐mediated Nrf2 activation increases the stress response and inhibits oxidative stress in osteoblasts in vitro, and also decreases cellular senescence and increases bone formation in vivo. In contrast, Nrf2‐deficient mice here exhibited severe aging‐related osteoporosis, with severe bone loss. These findings indicate that Nrf2 is not only essential for inhibiting oxidative stress‐induced osteoclast activity (59), but also maintains osteoblast cell survival to increase osteoblast activity in response to oxidative stress with aging. Interestingly, the effects of Nrf2 on bone seem to be different between young and aging mice in bone‐related cells. Based on previous studies (Sánchez‐de‐Diego et al., 2021; Yang, Zhang, et al., 2022) and the present study, Nrf2 exerts more protective effects in response to aging‐induced stress in osteoblasts, and targeted activation of Nrf2 in osteoblasts in aged mice is needed to test this hypothesis.

Here, we also detail a novel mechanism whereby PQQ stabilized Nrf2 via MCM3‐mediated disruption of the interaction between Keap1 and Nrf2. The modification of cysteine residues and conformational change in Keap1 can be induced by oxidative stress, reducing Nrf2 ubiquitination, and degradation. Additionally, mounting evidence indicates that there is cross‐talk between Keap1‐Nrf2 and other proteins, such as aPKCι, iASPP2, p62, and Nestin (Ge et al., 2017; Komatsu et al., 2010; Tian et al., 2019; Wang et al., 2019), which can disrupt the normal Keap1‐Nrf2 interaction and facilitate Nrf2‐mediated cellular redox homeostasis. Here, we found that PQQ preferably binds to MCM3 using a small molecule drug binding protein prediction site (http://www.lilab‐ecust.cn/pharmmapper/). We further used Autodocking software to verify the direct interaction between PQQ and MCM3. As a novel substrate of Keap1, MCM3 was reported to compete with Nrf2 for binding to Keap1, thus inhibiting Nrf2 degradation (Tamberg et al., 2018). Here, we found that PQQ inhibits MCM3 ubiquitination and proteasome degradation; furthermore, PQQ‐mediated Nrf2 stability was blocked following MCM3‐knockdown. Overall, these data provide a novel mechanism by which PQQ can inhibit the degradation of Nrf2 through the MCM3‐Keap1‐Nrf2 axis. However, our studies did not clarify how PQQ binding regulates MCM3 protein stability. A previous study has reported that Keap1 can directly ubiquitinates MCM3; however, Keap1 did not regulate total MCM3 protein stability and subcellular localization (Mulvaney et al., 2016). Therefore, the underlying molecule essential for MCM3 protein degradation needs to be further investigated, and this might be helpful to explain how PQQ binding regulates MCM3 protein stability.

Although previous studies have reported that Nrf2 is essential for osteoclast differentiation, whether Nrf2 can directly stimulate osteoclastic differentiation independent of oxidative stress‐induced Rankl production in osteoblasts is unclear. Here, we performed RNA‐seq assay and found that Fbn1 was significantly decreased in Nrf2^−/−^ BM‐MSCs apart from other known Nrf2 downstream targets. Fbn1 has been reported to reduce osteoclast activity in vitro and in vivo by antagonizing Rankl and inhibiting NFATc1 signaling (Smaldone et al., 2016; Tiedemann et al., 2013). Consistently, we found that Fbn1 knockdown in osteoblasts reduces osteoclast differentiation of mouse BMMs in the co‐culture system. Furthermore, we used a website (https://jaspar.genereg.net/) to predict possible binding sites of Nrf2 in the Fbn1 promoter, and found one classic ARE site in the Fbn1 promoter. We found that Nrf2 can transcriptionally upregulate Fbn1 by binding to its promoter, and this effect of Nrf2 on Fbn1 appears to be independent of oxidative stress regulation as the antioxidant NAC does not rescue the downregulation of Fbn1 caused by Nrf2 deficiency. In conclusion, we report here, for the first time, that Nrf2 can bind to the promoter region of Fbn1 to regulate Fbn1 expression and antagonize Rankl, providing a novel mechanism for Nrf2 activation in decreasing osteoclast differentiation.

To further determine whether Nrf2 could serve as a key target of PQQ in correcting natural aging‐induced osteoporosis, 6‐month‐old WT and Nrf2^−/−^ mice were supplemented with PQQ in vivo; osteoblasts isolated from WT and Nrf2^−/−^ mice were treated with PQQ, and their phenotypes were analyzed in vivo and in vitro. Here, we report that aged Nrf2‐deficient mice displayed severe aging‐related bone loss and a skeletal aging phenotype, although the effect of Nrf2 deficiency on bone in young mice was inconclusive in previous studies (Kim et al., 2014; Park et al., 2014; Yin et al., 2020). Moreover, we found that PQQ supplementation corrected the increased bone resorption and bone loss in aging WT mice; however, these responses were largely blocked in Nrf2^−/−^ mice. In addition, we found that PQQ inhibits oxidative stress in osteoblasts and decreased osteoclastogenesis in OB‐OC co‐culture systems; however, these responses were significantly blocked by Nrf2 deficiency. Overall, these results suggest that Nrf2 is essential for PQQ to alleviate aging‐induced osteoporosis by increasing an oxidative stress response and reducing Rankl production in osteoblasts.

In summary, the results of this study reveal that PQQ can bind to MCM3 and reduce its ubiquitination‐mediated degradation, thus stabilizing MCM3. MCM3 then competes with Nrf2 for binding to Keap1 and enhances Nrf2‐ARE signaling. PQQ‐induced Nrf2 activation in osteoblast‐lineage cells increases the cellular stress response, and also transcriptionally activates Fbn1, thus reducing the production of Rankl and inhibiting osteoclastic bone resorption, and preventing aging‐related osteoporosis. In addition, PQQ‐mediated Nrf2 activation in osteoblasts‐lineage cells can inhibit DNA damage and cellular senescence and increase osteoblastic bone formation. These findings suggest that PQQ could be an ideal supplement for the treatment and prevention of aging‐related osteoporosis.

## AUTHOR CONTRIBUTIONS

Renlei Yang and Dengshun Miao conceived the project. Jie Li and Jing Zhang performed most of the experiments, analyzed, and compiled the data. Qi Xue, Boyang Liu, Ran Qin, Yiping Li, Yue Qiu, and Rong Wang helped with experiments. Renlei Yang, Dengshun Miao, and David Goltzman participated in writing or editing the paper.

## CONFLICT OF INTEREST STATEMENT

The authors declare no competing interests.

## Supporting information


Data S1.

## Data Availability

All data and materials used in the study are available to any researcher for purposes of reproducing or extending the analysis.
