# Supplementary material for: Pyrroloquinoline quinone alleviates natural aging‐related osteoporosis via a novel MCM3‐Keap1‐Nrf2 axis‐mediated stress response and Fbn1 upregulation
Source: Aging Cell. 2023 Jun 26;22(9):e13912. doi: 10.1111/acel.13912 (PMC10497824; doi:10.1111/acel.13912)

**Supporting Information**


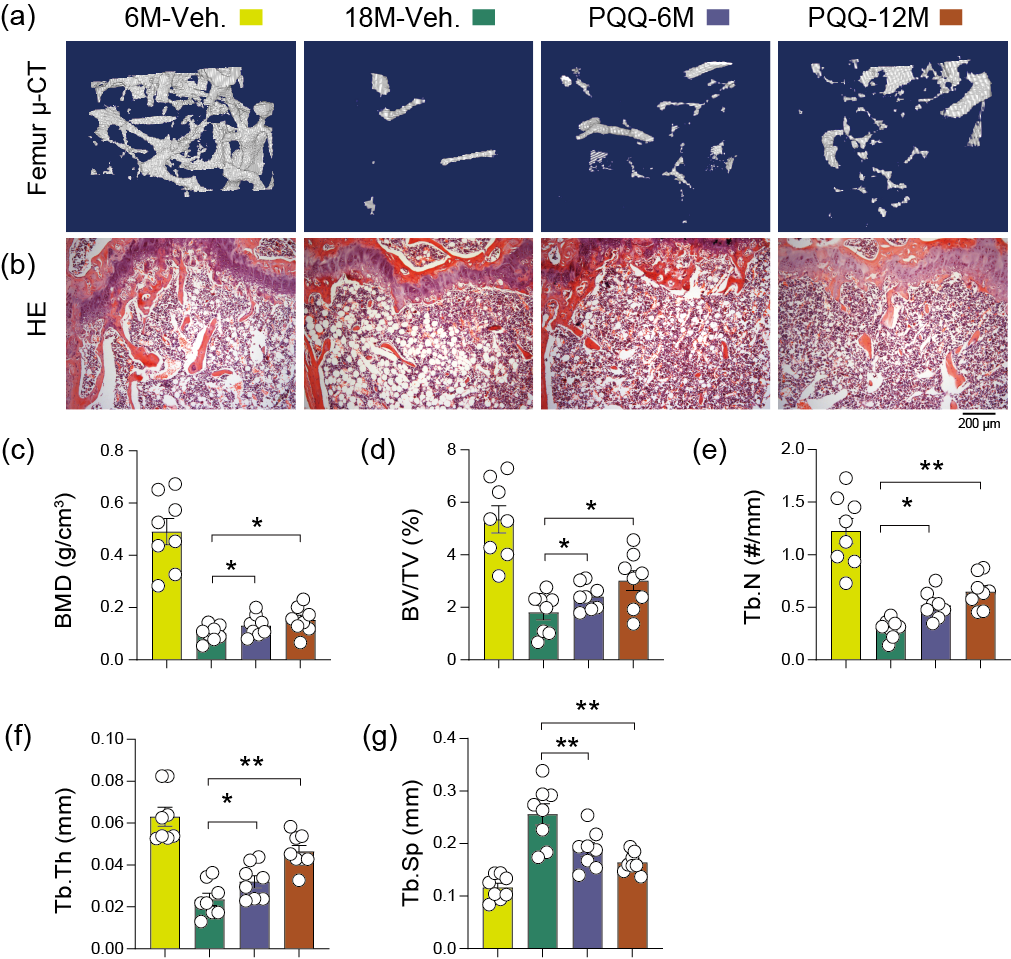
**Supplementary file 1**

**Fig. S1. Micro-CT and HE staining of femurs in indicated groups of mice.**

(a) Representative μCT scans and (b) HE staining of femurs from indicated groups of mice. Microtomography indices were measured as (c) BMD, (d) BV/TV, (e) Tb.N, (f) Tb.Th and (g) Tb.Sp. One-way ANOVA. *: p < 0.05, **: p < 0.01.


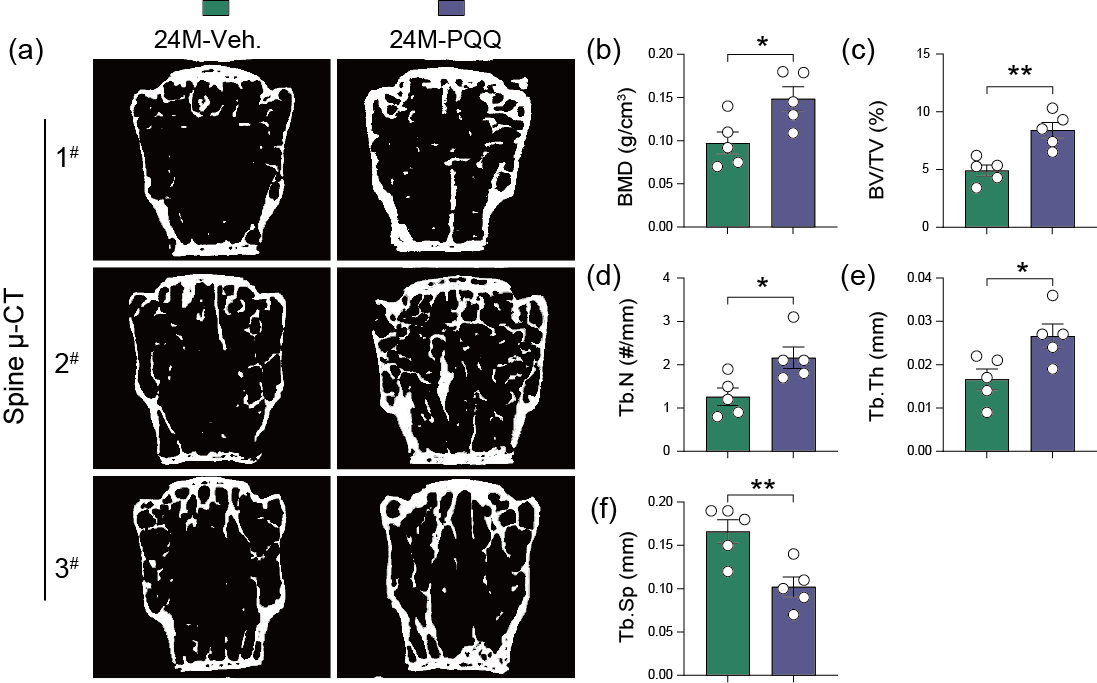


**Fig. S2. Micro-CT images of** **lumbar vertebrae and microtomography indices.**

(a) Representative μCT scans of lumbar vertebrae in 24-month-old wild-type mice treated with vehicle or PQQ for 12 months. Microtomography indices were measured as (b) BMD, (c) BV/TV, (d) Tb.N, (e) Tb.Th and (f) Tb.Sp. Two-tailed Student’s t test. *: p < 0.05, **: p < 0.01.


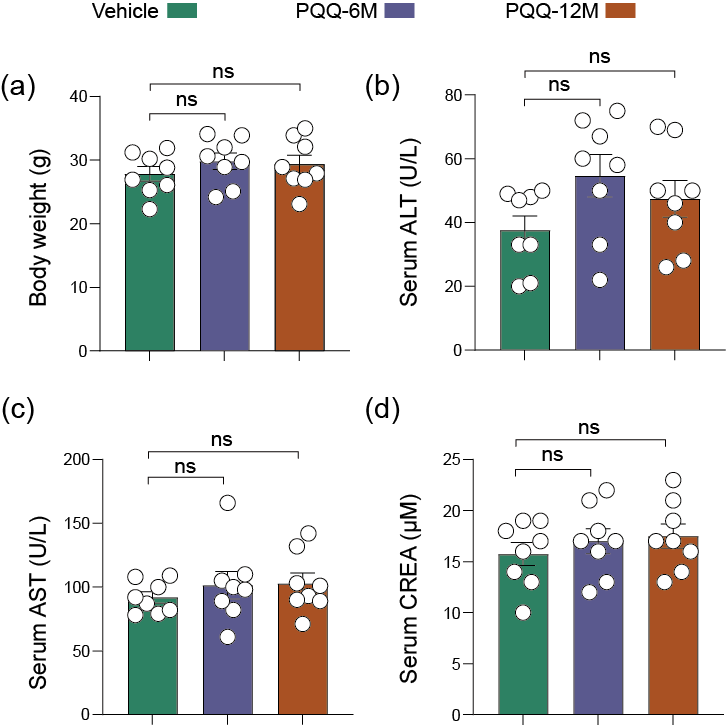


**Fig. S3. Body weight, serum ALT, AST and CERA levels upon PQQ supplementation in aged mice.**

(a) Body weight, and serum (b) ALT, (c) AST and (d) CERA levels in indicated groups of mice. One-way ANOVA. ns: not significant.


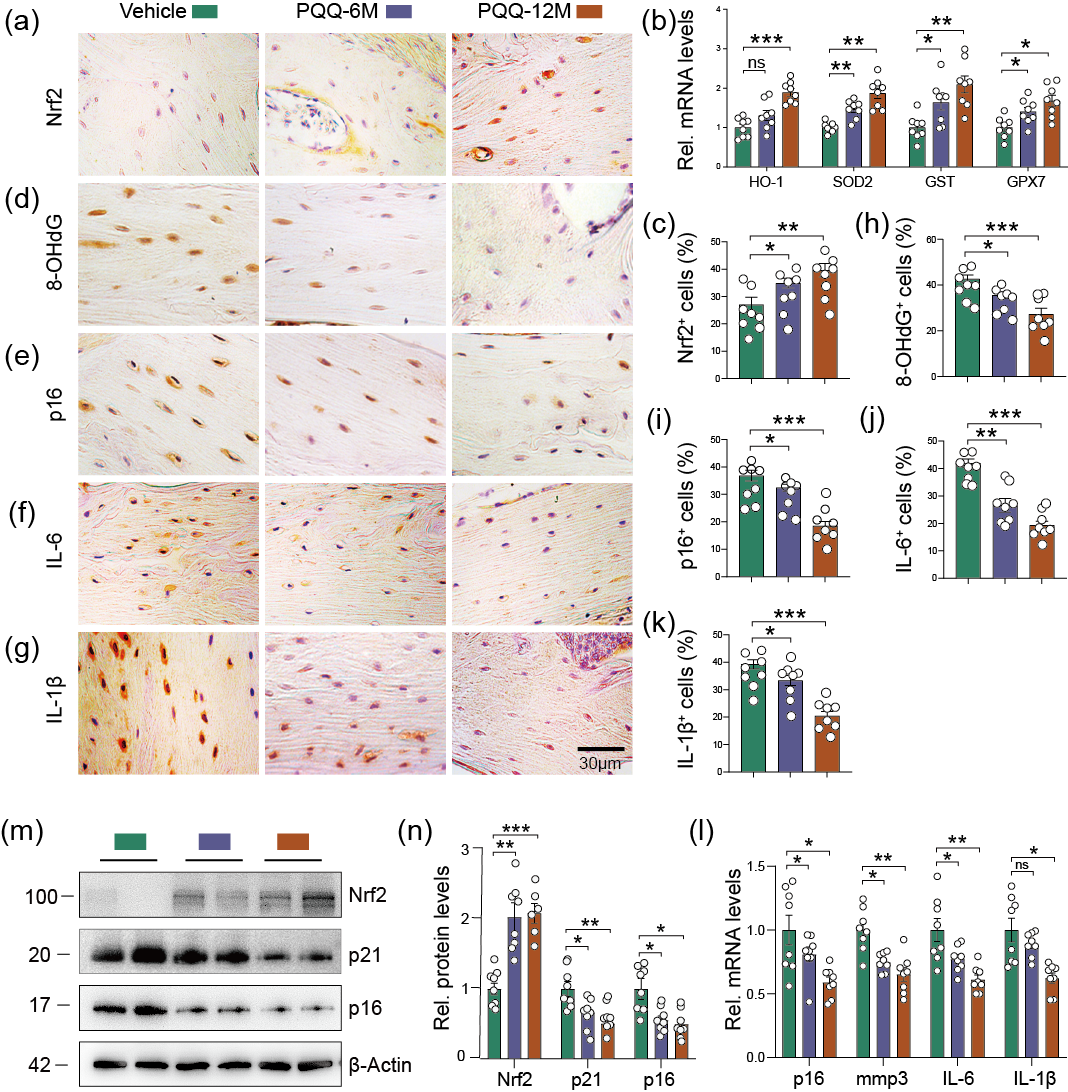


**Fig. S4. PQQ supplementation alleviates bone aging phenotype in aged wild-type mice.**

(a) Representative micrographs of vertebral cortical sections immunostained for Nrf2. (b) Real-time PCR detection of antioxidant-related genes heme oxygenase-1 (HO-1), glutathione S-transferases (GST), SOD2 and glutathione peroxidase7 (Gpx7) in vertebral tissues of indicated groups of mice. (c) Quantification of the percentage of Nrf2^+^ osteocytes. (d-k) Representative micrographs of vertebral cortical sections immunostained for (d) DNA damage marker 8-OHdG, (e) p16, (f) IL-6 and (g) IL-1β. Quantification of the percentages of (h) 8-OHdG^+^, (i) p16^+^, (j) IL-1β^+^ and (k) IL-6^+^ osteocytes. (l) Relative mRNA levels of p16, matrix metalloproteinase-3 (Mmp3), IL-6 and IL-1β in bony tissues from indicated groups of mice. (m) Western blot detection of Nrf2, p21 and p16 in indicated bone tissues, and (n) the quantitative protein analysis. One-way ANOVA. *: p < 0.05, **: p < 0.01, ***: p < 0.001. ns: not significant.


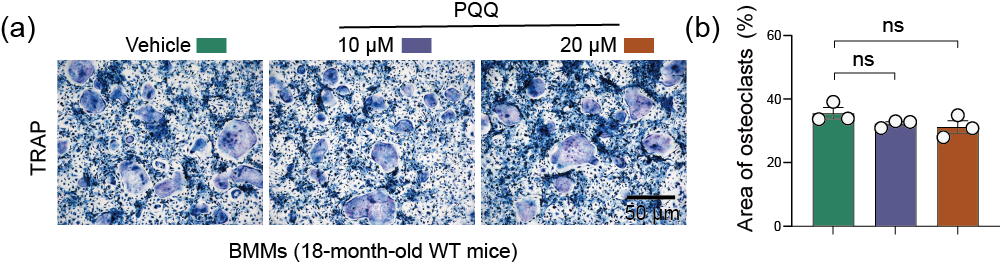


**Fig. S5. The effect of PQQ on osteoclast differentiation of BMMs isolated from aged mice.**

(a) BMMs of 18-month-old wild-type mice were treated with indicated concentrations of PQQ for 48 hours, after which BMMs were treated with Rankl and M-CSF to induce osteoclast differentiation determined using TRAP staining. (b) Quantitative analysis of the area of osteoclasts. Two-tailed Student’s t test. ns: not significant.


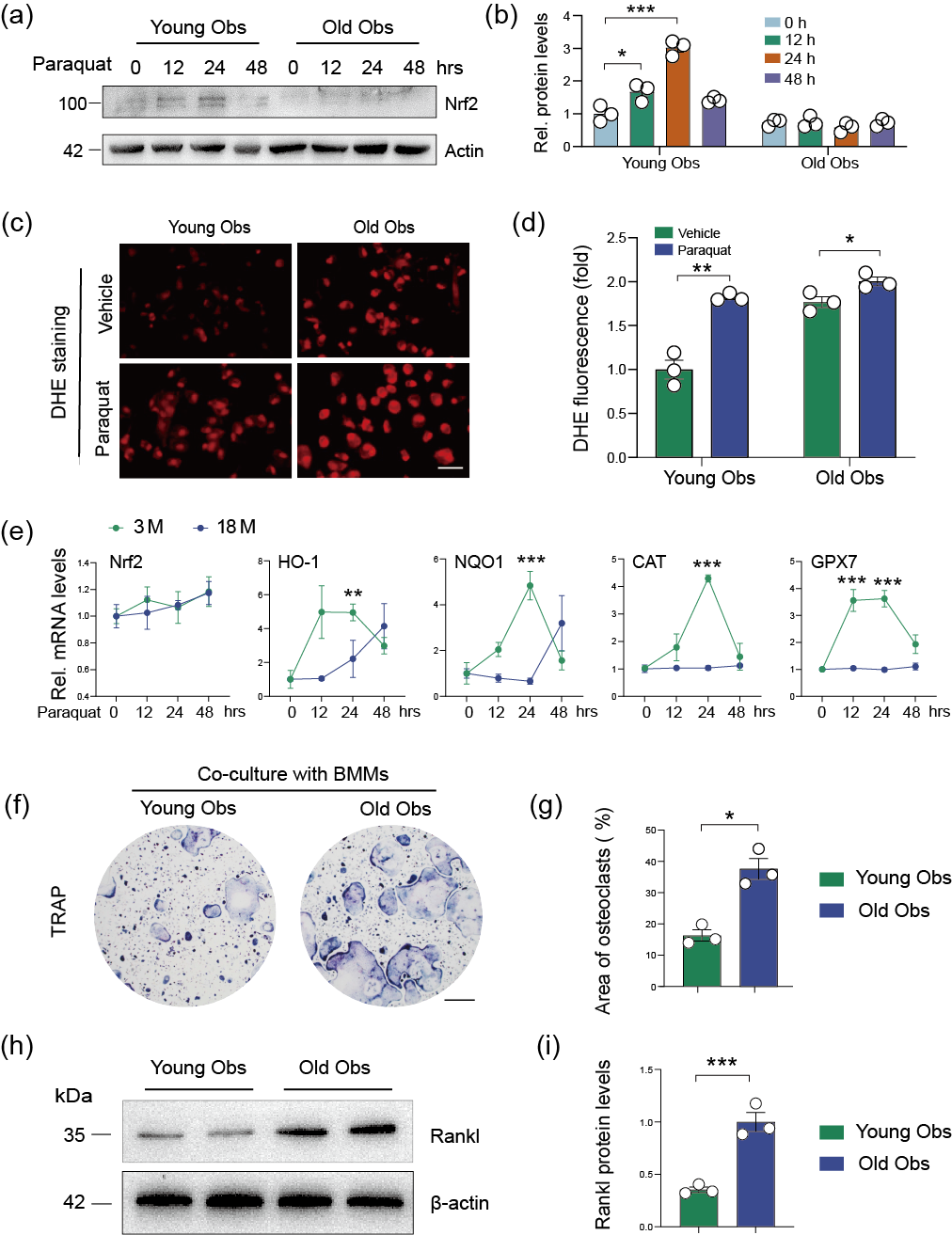


**Fig. S6. Senescent osteoblasts display decreased stress response capacity and increased Rankl production due to Nrf2 downregulation.**

(a) Western blot detection of Nrf2 protein levels in osteoblasts from 3- and 18-month-old wild-type mice in response to paraquat treatment for indicated times. (b) Quantitative analysis of (A). (c) ROS levels in indicated groups of osteoblasts treated with vehicle or paraquat were detected using DHE staining. (d) Quantitative analysis of (c). (e) Real-time PCR detection of Nrf2, HO1, Nqo1, CAT and GPX7 mRNA levels in osteoblasts from 3- and 18-month-old wild-type mice treated with vehicle or paraquat for indicated times. Two-way ANOVA. *: p < 0.05, **: p < 0.01, ***: p < 0.001. (f) Osteoclastogenesis by OB-OC co-culture in vitro using osteoblasts from 3- and 18-month-old wild-type mice and (g) the quantitative analysis of the area of TRAP-positive multinucleated cells. (h) Western blot detection of Rankl in osteoblasts from 3- and 18-month-old wild-type mice and (i) the quantification of Rankl protein levels. Two-tailed Student’s t test. *: p < 0.05, ***: p < 0.001.


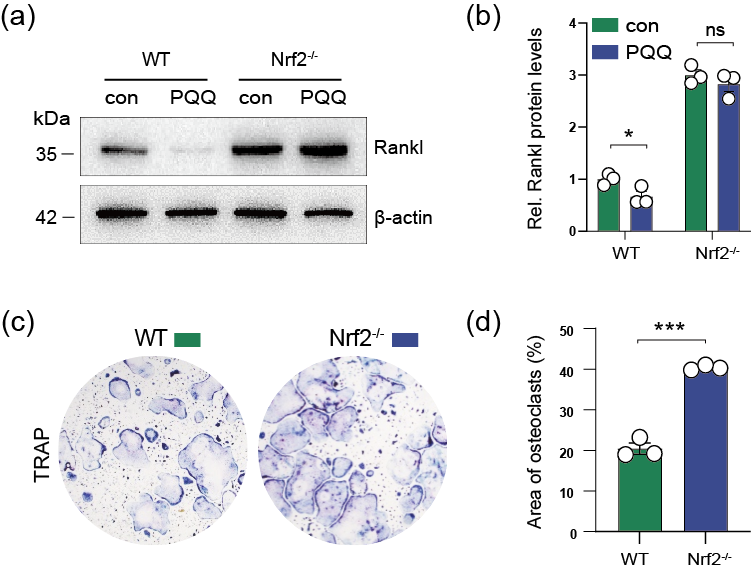


**Fig. S7. Nrf2-deficient osteoblasts lead to higher Rankl production and increased osteoclastogenesis.**

(a) Western blot detection and (b) the quantitative analysis of Rankl protein levels in indicated groups. (c) WT and Nrf2-deficient osteoblasts were co-cultured with BMMs from wild-type mice in the presence of 1,25(OH)_2_D_3_ and PGE2, and then osteoclasts were detected using TRAP staining. (d) Quantitative analysis of the area of osteoclasts. Two-tailed Student’s t test. *: p < 0.05, ***: p < 0.001. ns: not significant.


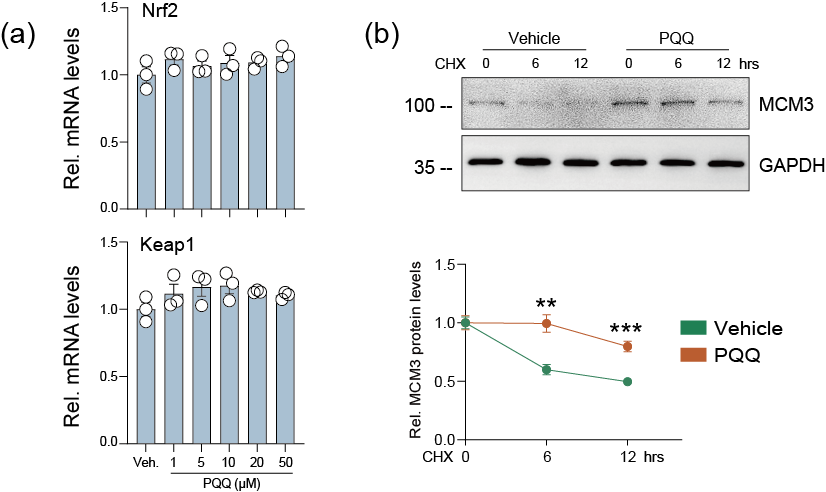


**Fig. S8. The effects of PQQ on the transcription of Nrf2 and Keap1, and on the protein degradation of MCM3.**

(a) The mRNA levels of Nrf2 and Keap1 in hBM-MSCs treated with vehicle or indicated dose of PQQ were determined using qPCR. (b) Western blot detection of MCM3 protein levels in vehicle- or PQQ-treated hBM-MSCs in the absence and presence of CHX for indicated times. Two-way ANOVA. **: p < 0.01, ***: p < 0.001.


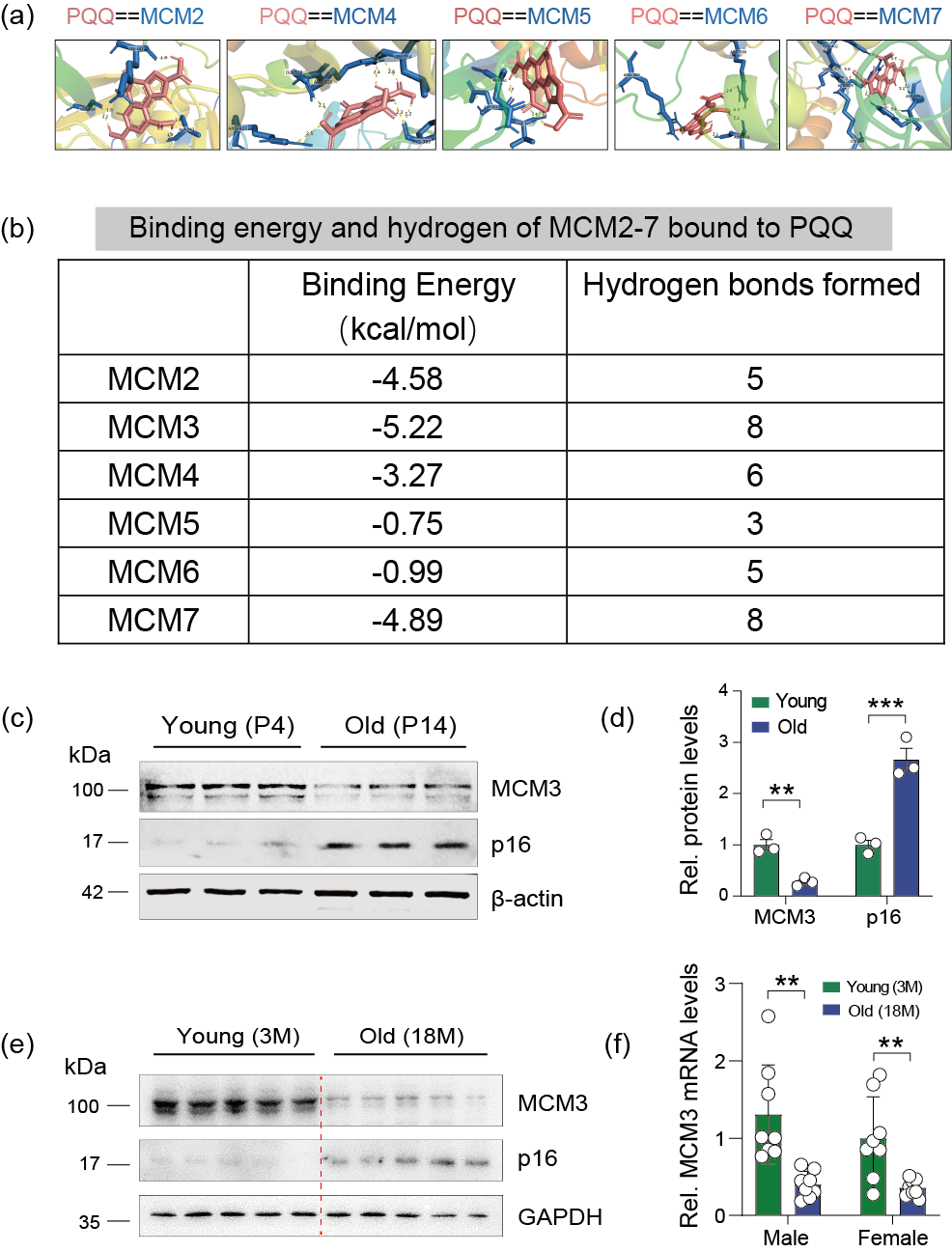


**Fig. S9. PQQ preferably binds to MCM3 in the MCM family, and MCM3 expression is significantly reduced with aging.**

(a) Molecular docking results of PQQ with MCM subunits 2, 4, 5, 6 and 7. (b) Binding energy and the number of hydrogen bonds for the docking of PQQ and indicated MCM subunits 2-7. (c) MCM3 protein levels in young (passage 4) and old (passage 14) human BM-MSCs. (d) Quantitative analysis of (c). (e-f) MCM3 protein levels in young (3-month-old) and old (18-month-old) mice determined using Western blot and qPCR. Two-tailed Student’s t test. **: p < 0.01, ***: p < 0.001.


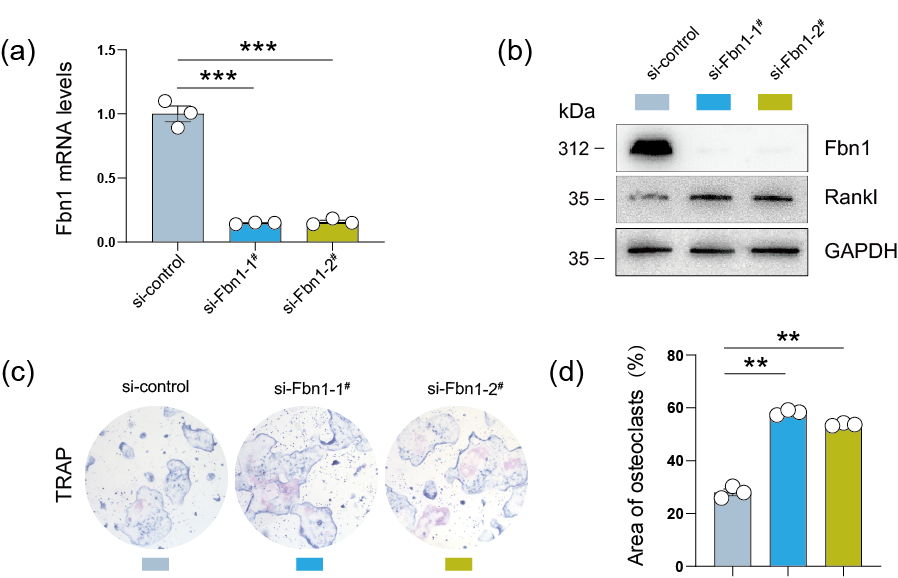


**Fig. S10. Fbn1 knockdown in osteoblasts increases Rankl production in osteoblasts and promotes osteoclast formation in the OB-OC co-culture system.**

(a) qPCR detection of Fbn1 and (b) Western blot detection of Fbn1 and Rankl in mouse osteoblasts following si-Fbn1 treatment. (c) Control and Fbn1-knockdown osteoblasts were co-cultured with wild-type BMMs for osteoclast differentiation in the presence of 1,25(OH)_2_D_3_ and PGE2, and osteoclasts were detected using TRAP staining. (d) Quantitative analysis of the area of osteoclasts in (c). Two-tailed Student’s t test. *: p < 0.05, **: p < 0.01, ***: p < 0.001.


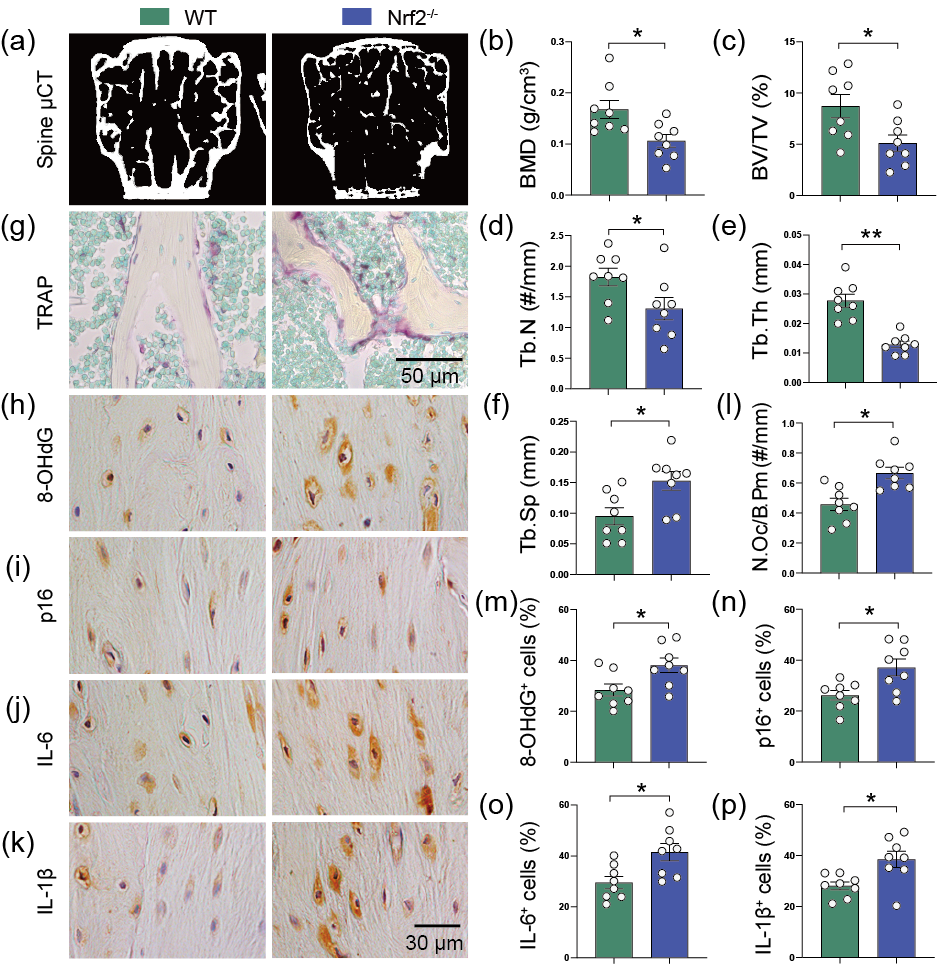


**Fig. S11. Nrf2 deficiency accelerates aging-related bone loss.**

Skeletal phenotypes of WT and Nrf2^-/-^ mice were analyzed at the age of 18 months. (a) Representative μCT scans of 3D longitudinal reconstructions. Microtomography indices were evaluated as (b) bone mineral density (BMD), (c) trabecular bone volume (BV/TV), (d) trabecular number (Tb.N), (e) trabecular thickness (Tb.Th) and (f) trabecular separation (Tb.Sp). (g) Representative micrographs of vertebral trabecular sections stained histochemically for TRAP and (l) the quantitative analysis of the number of osteoclasts per bone perimeter (N.Oc/B.Pm, #/mm). Representative micrographs of vertebral cortical sections immunostained for (h) DNA damage marker 8-OHdG, (i) p16, (j) IL-6 and (k) IL-1β. Quantification for the percentages of (m) 8-OHdG^+^, (n) p16^+^, (o) IL-6^+^ and (p) IL-1β^+^ osteocytes. Two-tailed Student’s t test. *: p < 0.05, **: p < 0.01, ***: p < 0.001.

**Supplementary file 2**

**Supplementary Table 1. Primers used for ChIP-PCR**

|  | Forward | Reverse |
| --- | --- | --- |
| Fbn1-Site1  Fbn1-Site2 | CTTTTGCTCTTTCTAAGTCGCCA AACCCTTACTGGGGTTTGCT | ACATGCCAACAAACACGGG ATGTGATCCTGTCCCCTGTC |

**Supplementary Table 2. Primers used for quantitative real-time PCR**

|  | Species | Forward | Reverse |
| --- | --- | --- | --- |
| Gapdh | Human | ACAACTTTGGTATCGTGGAAGG | GCCATCACGCCACAGTTTC |
| Nrf2 | Human | TTCTGTTGCTCAGGTAGCCCC | TCAGTTTGGCTTCTGGACTTGG |
| Keap1 | Human | GTGTCCATTGAGGGTATCCACC | GCTCAGCGAAGTTGGCGAT |
| MCM3 | Human | TCAGAGAGATTACCTGGACTTCC | TCAGCCGGTATTGGTTGTCAC |
| Gapdh | Mouse | AGGTCGGTGTGAACGGATTTG | TGTAGACCATGTAGTTGAGGTCA |
| Nrf2 | Mouse | TCTTGGAGTAAGTCGAGAAGTGT | GTTGAAACTGAGCGAAAAAGGC |
| HO1 | Mouse | CACGCATATACCCGCTACCT | CCAGAGTGTTCATTCGAG |
| Nqo1 | Mouse | AGGATGGGAGGTACTCGAATC | AGGCGTCCTTCCTTATATGCTA |
| Gpx7 | Mouse | GGGGTGACGAGGTGGAGTA | GTTGGGGTTTGTCCTCTCCC |
| MCM3 | Mouse | AGCGCAGAGAGACTACTTGGA | CAGCCGATACTGGTTGTCACT |
| Keap1 | Mouse | TGCCCCTGTGGTCAAAGTG | GGTTCGGTTACCGTCCTGC |
| Opg | Mouse | ACCCAGAAACTGGTCATCAGC | CTGCAATACACACACTCATCACT |
| Rankl  CAT | Mouse  Mouse | CAGCATCGCTCTGTTCCTGTA AGCGACCAGATGAAGCAGTG | CTGCGTTTTCATGGAGTCTCA TCCGCTCTCTGTCAAAGTGTG |
| Fbn1 | Mouse | GGACGCCAATTTGGAGGCT | CTTTCAGCGCATCGTGTCCT |
| P16 | Mouse | CGCAGGTTCTTGGTCACTGT | TGTTCACGAAAGCCAGAGCG |
| Mmp3 | Mouse | ACATGGAGACTTTGTCCCTTTTG | TTGGCTGAGTGGTAGAGTCCC |
| IL-6 | Mouse | TAGTCCTTCCTACCCCAATTTCC | TTGGTCCTTAGCCACTCCTTC |
| IL-1β | Mouse | GCAACTGTTCCTGAACTCAACT | ATCTTTTGGGGTCCGTCAACT |

**Supplementary Table 3. Indicated promoter sequences of Fbn1 cloned to pGL4.17**

| **Fbn1 promoter-** **pGL4.17** |
| --- |
| GTGGGGATCTTTGAAAAATAGCCATGTCAATGTCCACCACAGACTTTAAAAAACTGGGTCCTTGCCTATTTCTTTTTAAGTTCCTAGAACTTACTGTGTATCTATAGTTAAGAACTTTTGCTCTTTCTAAGTCGCCATGGTGCCTTAAAAGGGTTCAATGATACTTTATCAGGGAAGCCTCCTCTATTGGCCCTAGCCTATGTCTAGTCATGGTCACATACATCATCTAAGCAGTGCCCCGTGTTTGTTGGCATGTAACATATGAGCTACTATTTGTTTCGGAGGTGAAATAAAGTCTCCTGGGTTTAAAGTATCCCTGCTTTGTTATCTGTGGCTTCTGTGTATGTGTGATGTAAGCACCAGGAGAAGAAATCACATCTTCAGTTAGTCCCTTCACGGTGGTACCGATTGCTGA |
| **Fbn1 promoter mutant- pGL4.17** |
| GTGGGGATCTTTGAAAAATAGCCATGTCAATGTCCACCACAGACTTTAAAAAACTGGGTCCTTGCCTATTTCTTTTTAAGTTCCTAGAACTTACTGTGTATCTATAGTTAAGAACTTTTGCTCTTTCTAAGTCGCCATGGTGCCTTAAAAGGGTTCAATGATACTTTATCAGGGAAGCCTCCTCTATTGGCCCTAGCCTAACATACATCATCTAAGCAGTGCCCCGTGTTTGTTGGCATGTAACATATGAGCTACTATTTGTTTCGGAGGTGAAATAAAGTCTCCTGGGTTTAAAGTATCCCTGCTTTGTTATCTGTGGCTTCTGTGTATGTGTGATGTAAGCACCAGGAGAAGAAATCACATCTTCAGTTAGTCCCTTCACGGTGGTACCGATTGCTGA |

**Supplementary Table 4. Top 100 potential targets of PQQ predicted by PharmMapper.**


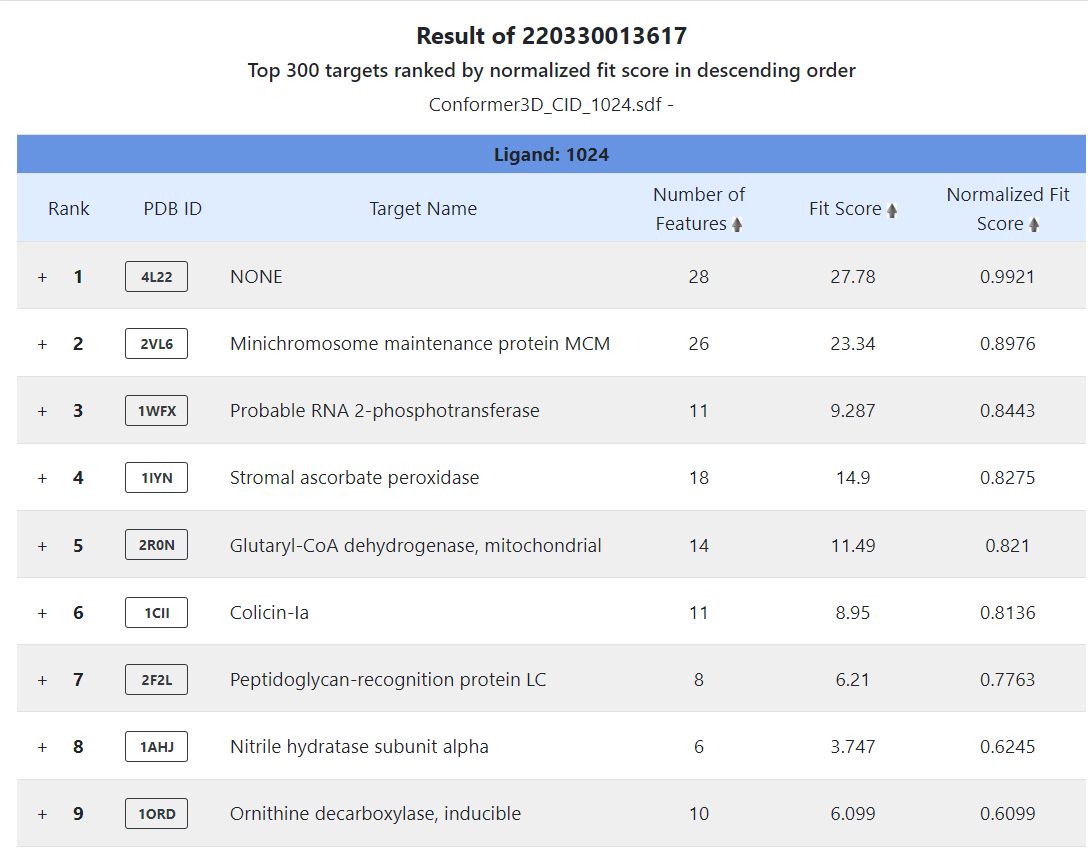


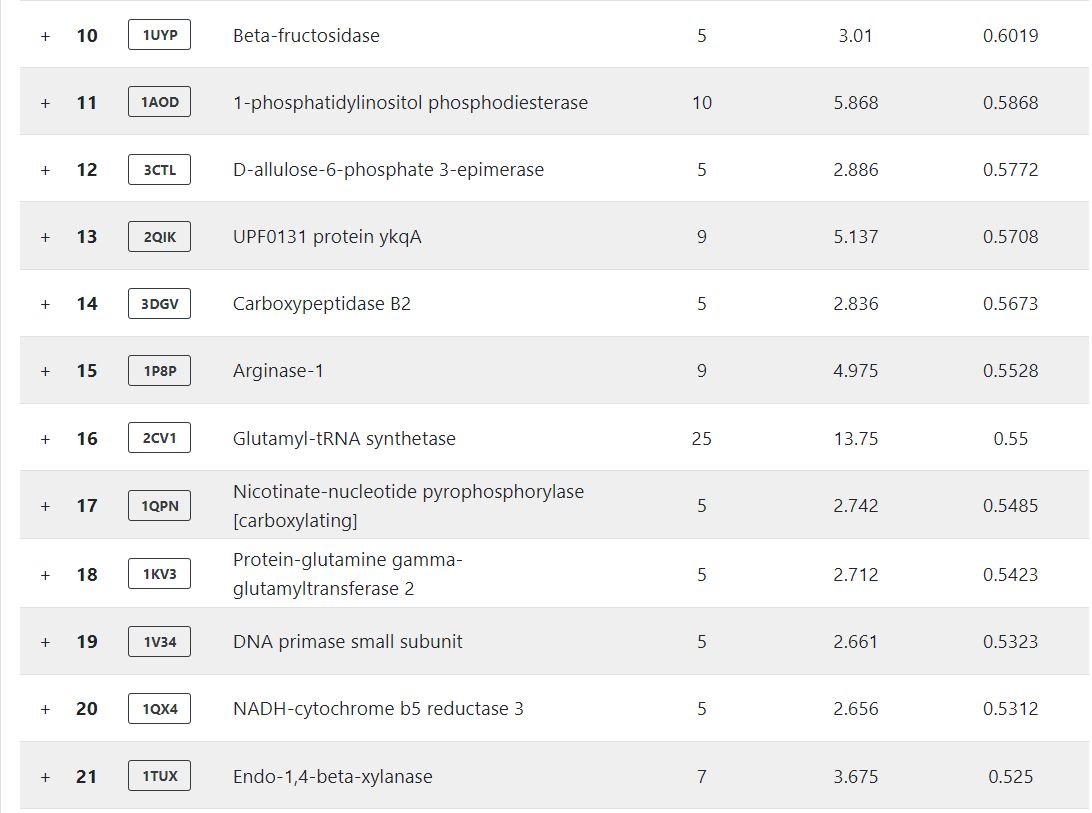


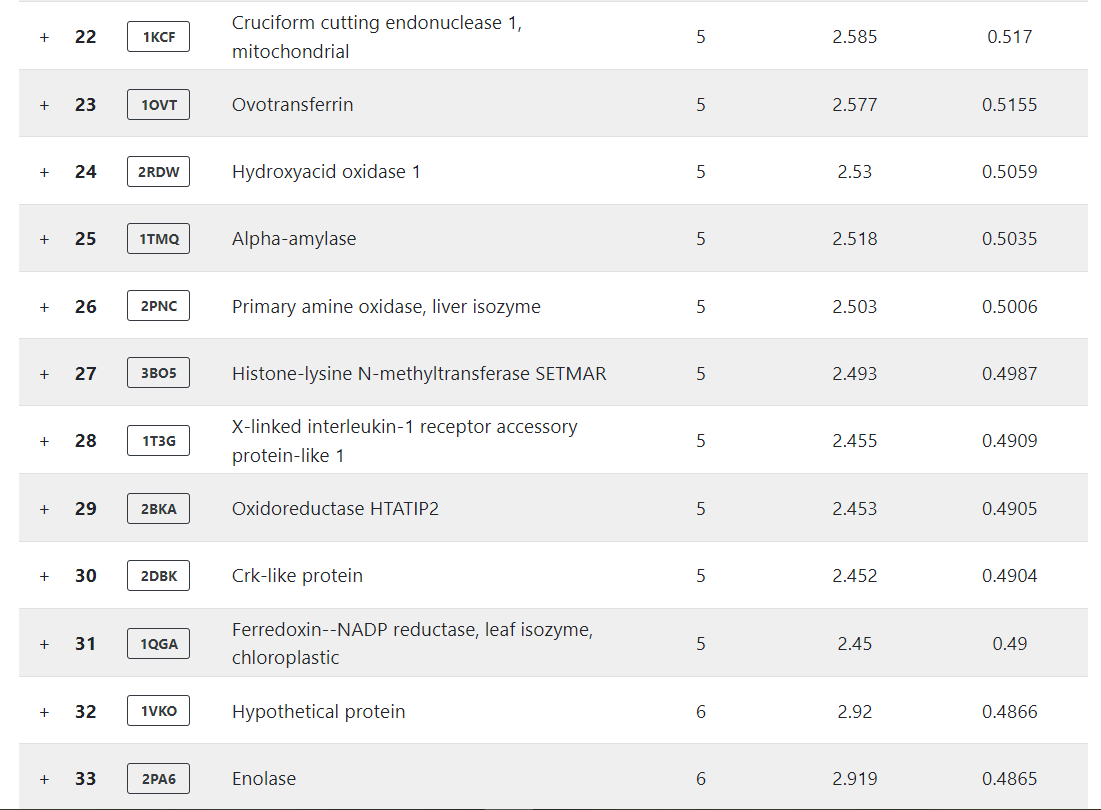

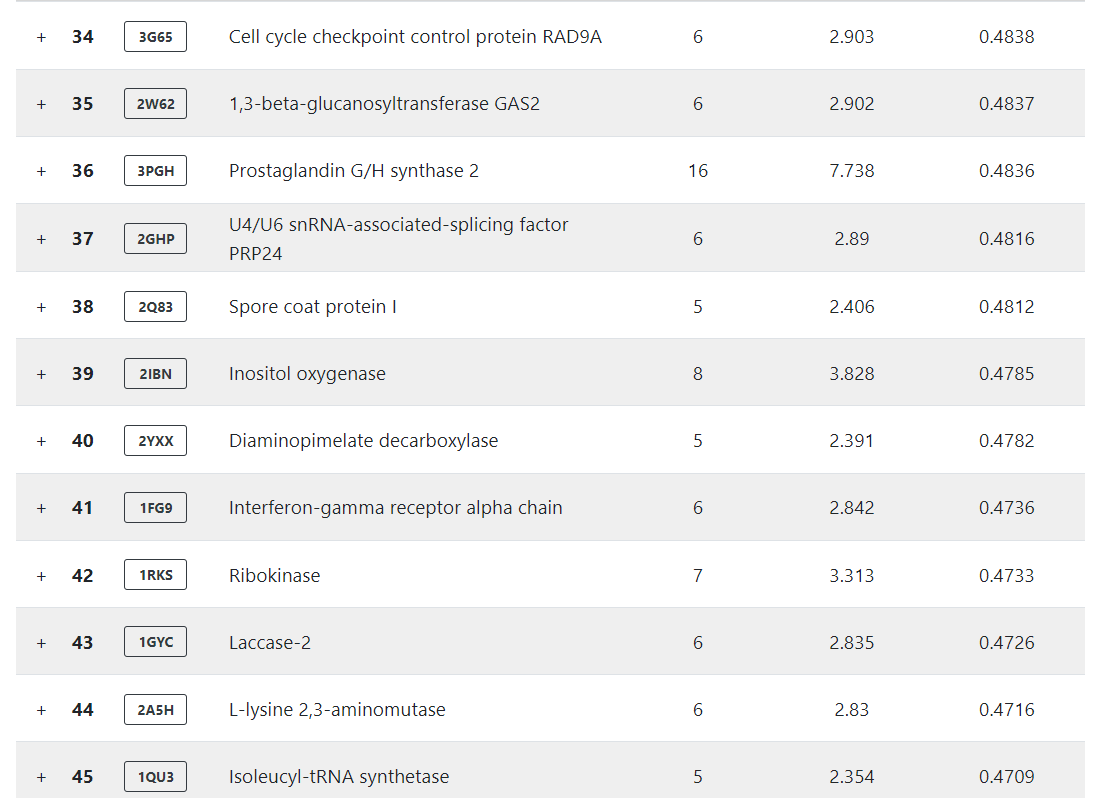


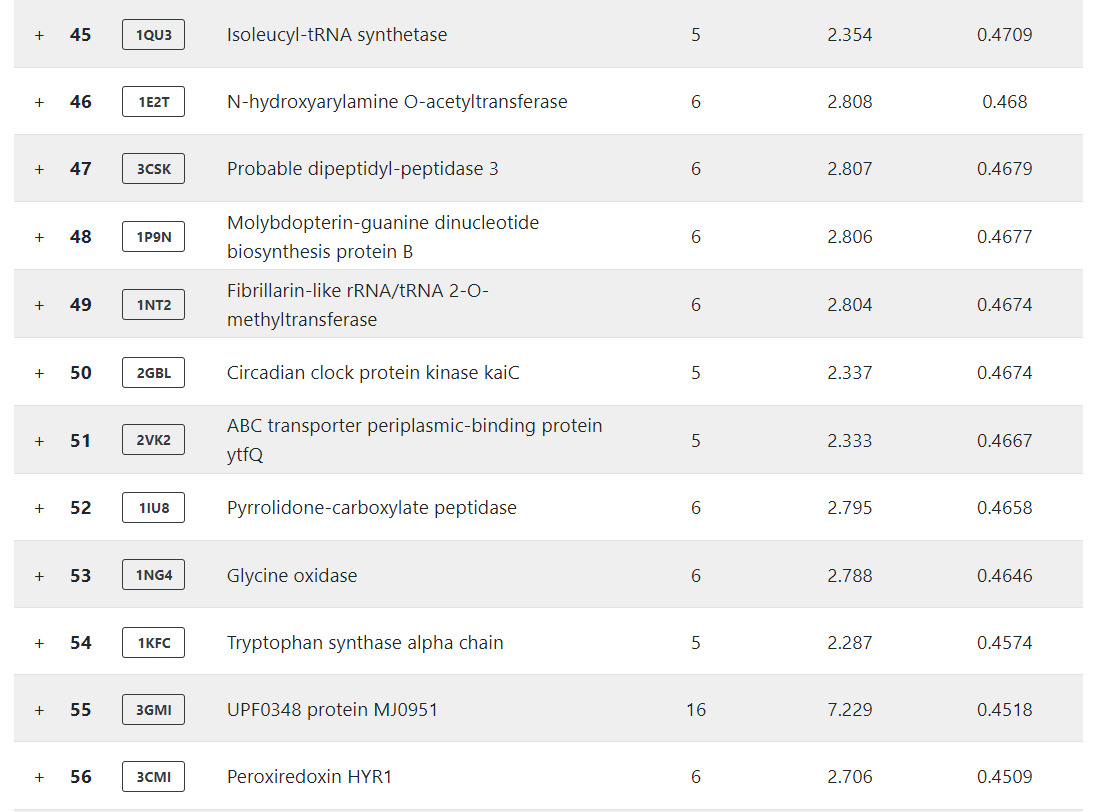


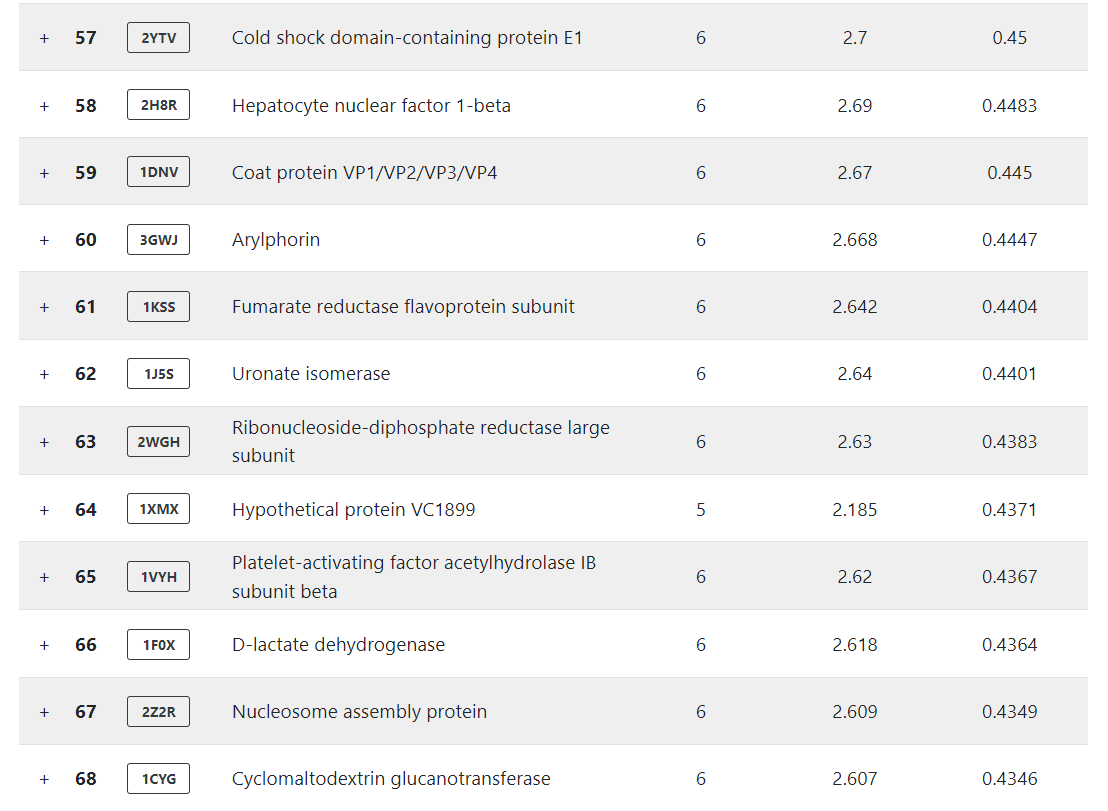


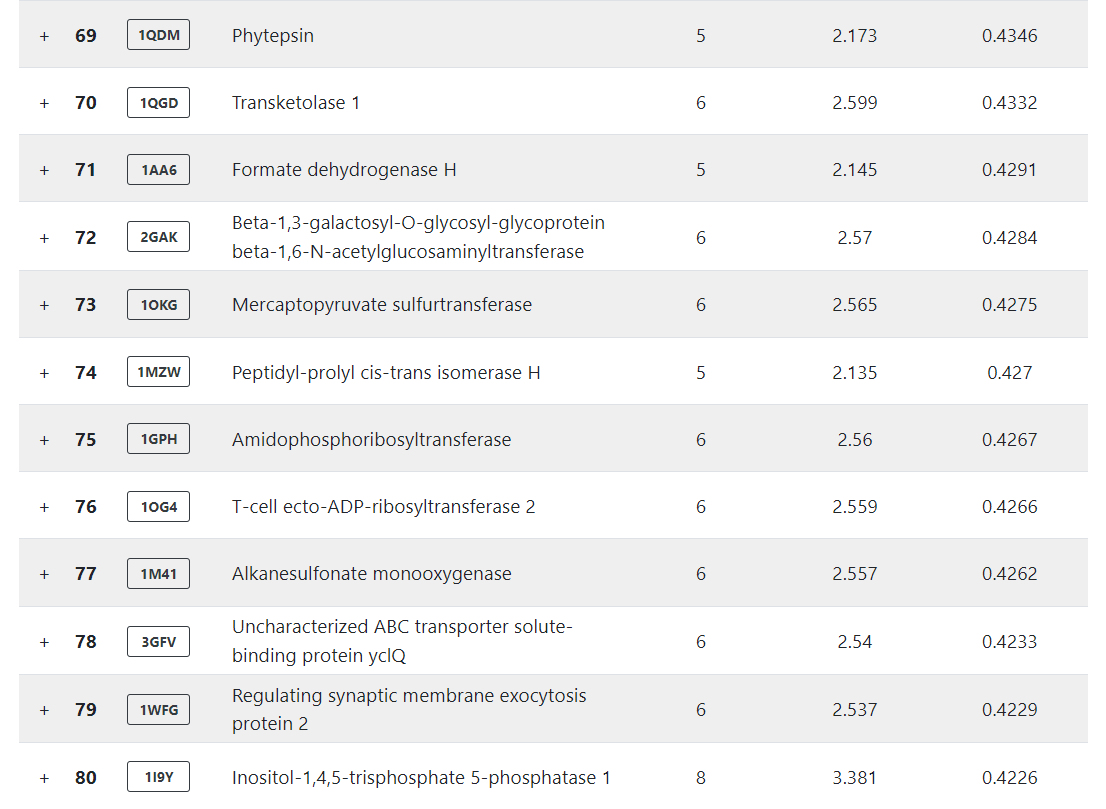


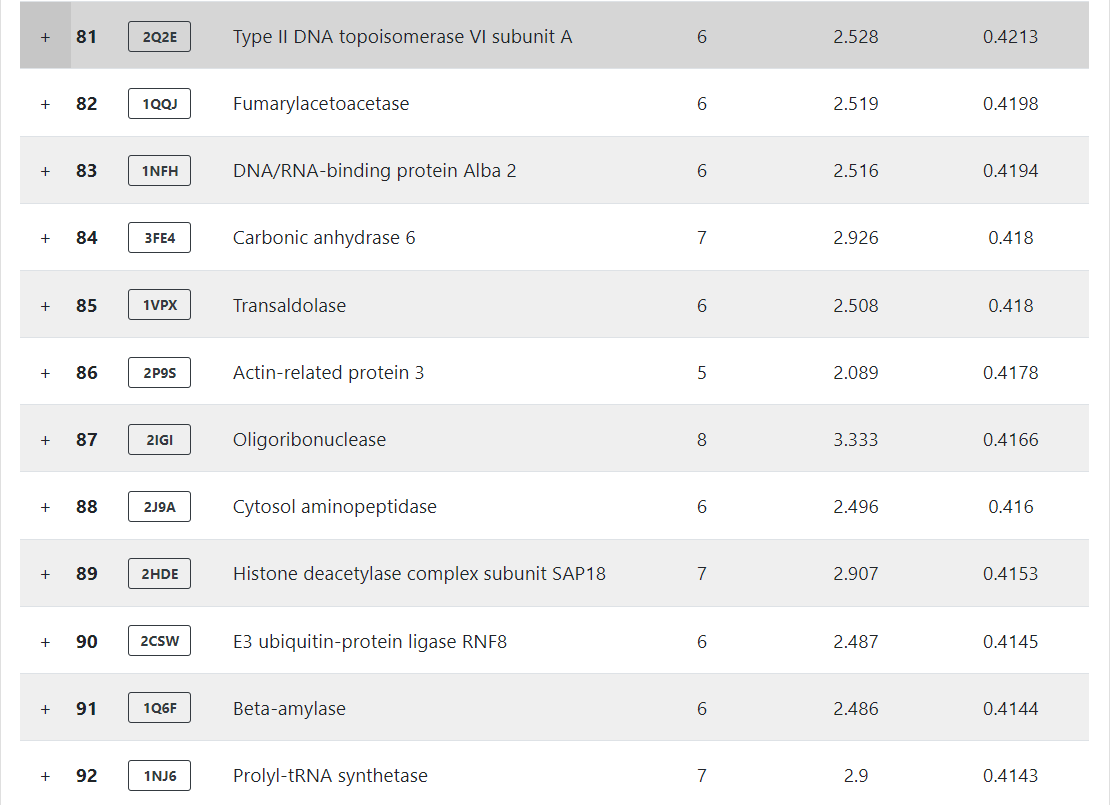


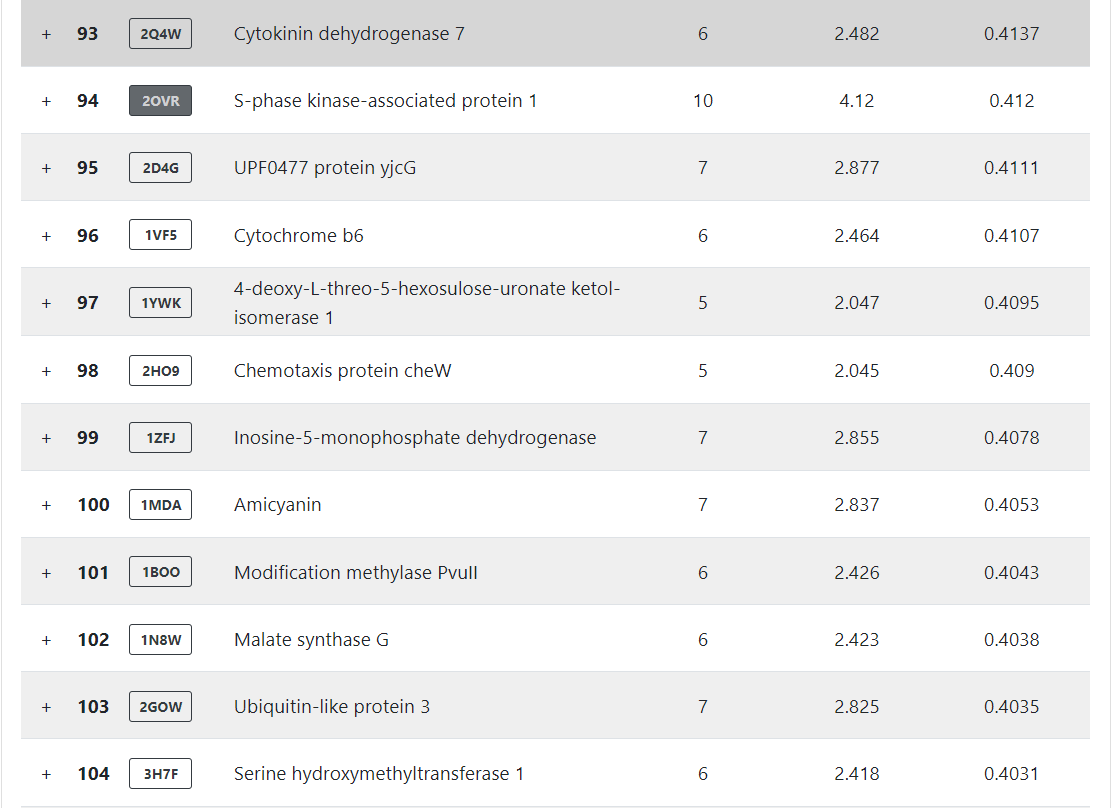

Supplement: Supplementary file 1 — Data S1. [file ACEL-22-e13912-s001.docx]
